# The pangenome of the wheat pathogen *Pyrenophora tritici-repentis* reveals novel transposons associated with necrotrophic effectors *ToxA* and *ToxB*

**DOI:** 10.1186/s12915-022-01433-w

**Published:** 2022-10-24

**Authors:** Ryan Gourlie, Megan McDonald, Mohamed Hafez, Rodrigo Ortega-Polo, Kristin E. Low, D. Wade Abbott, Stephen E. Strelkov, Fouad Daayf, Reem Aboukhaddour

**Affiliations:** 1 Agriculture and Agri-Food Canada, Lethbridge, AB Canada; 2 School of Biosciences, University of Birmingham, Institute of Microbiology and Infection, Edgbaston, Birmingham, UK; 3 Faculty of Agricultural, Life, and Environmental Sciences, University of Alberta, Edmonton, AB Canada; 4 Faculty of Agricultural and Food Sciences, University of Manitoba, Winnipeg, MB Canada

**Keywords:** Tan spot, Fungal wheat pathogen, Pangenome, ToxA, ToxB, Transposons, Fungal evolution, Necrotrophic effectors, Starship

## Abstract

**Background:**

In fungal plant pathogens, genome rearrangements followed by selection pressure for adaptive traits have facilitated the co-evolutionary arms race between hosts and their pathogens. *Pyrenophora tritici-repentis* (Ptr) has emerged recently as a foliar pathogen of wheat worldwide and its populations consist of isolates that vary in their ability to produce combinations of different necrotrophic effectors. These effectors play vital roles in disease development. Here, we sequenced the genomes of a global collection (40 isolates) of Ptr to gain insights into its gene content and genome rearrangements.

**Results:**

A comparative genome analysis revealed an open pangenome, with an abundance of accessory genes (~ 57%) reflecting Ptr’s adaptability. A clear distinction between pathogenic and non-pathogenic genomes was observed in size, gene content, and phylogenetic relatedness. Chromosomal rearrangements and structural organization, specifically around effector coding genes, were detailed using long-read assemblies (PacBio RS II) generated in this work in addition to previously assembled genomes. We also discovered the involvement of large mobile elements associated with Ptr’s effectors: *ToxA*, the gene encoding for the necrosis effector, was found as a single copy within a 143-kb ‘Starship’ transposon (dubbed ‘Horizon’) with a clearly defined target site and target site duplications. ‘Horizon’ was located on different chromosomes in different isolates, indicating mobility, and the previously described ToxhAT transposon (responsible for horizontal transfer of *ToxA*) was nested within this newly identified Starship. Additionally, *ToxB*, the gene encoding the chlorosis effector, was clustered as three copies on a 294-kb element, which is likely a different putative ‘Starship’ (dubbed ‘Icarus’) in a ToxB-producing isolate. *ToxB* and its putative transposon were missing from the *ToxB* non-coding reference isolate, but the homolog *toxb* and ‘Icarus’ were both present in a different non-coding isolate. This suggests that *ToxB* may have been mobile at some point during the evolution of the Ptr genome which is contradictory to the current assumption of *ToxB* vertical inheritance. Finally, the genome architecture of Ptr was defined as ‘one-compartment’ based on calculated gene distances and evolutionary rates.

**Conclusions:**

These findings together reflect on the highly plastic nature of the Ptr genome which has likely helped to drive its worldwide adaptation and has illuminated the involvement of giant transposons in facilitating the evolution of virulence in Ptr.

**Supplementary Information:**

The online version contains supplementary material available at 10.1186/s12915-022-01433-w.

## Background

*Pyrenophora tritici-repentis* (Ptr) is an ascomycete fungus that causes tan spot, one of the most destructive foliar diseases of wheat worldwide [1, 2]. Tan spot was not considered a significant disease of wheat until the 1970s, and from an evolutionary perspective, this recent emergence offers an intriguing case to gain insights into how a weak pathogen rose to a global threat in a short time span [2]. The Ptr genome is a mosaic of present and absent effectors and hence serves as a model for examining the evolutionary processes behind the acquisition of virulence in necrotrophs and how disease emergence develops.

In a fungal species, the genomic variation in contents and structural organization reflects its evolution and the selection pressures driving its diversification and speciation. Genome rearrangements, such as chromosomal polymorphisms, duplications, deletions, inversions, and fusions are very common in fungi. These rearrangements in plant pathogens can facilitate the selection for adaptive traits that enhance pathogenic abilities on a certain host in a certain niche, as the genome plasticity often will lead to the gain or loss of functions in the co-evolution arms race between the pathogen and its host. In this study, we took advantage of a diverse collection of Ptr isolates representing all pathotypes, from diverse global origins, and applied short- and long-read sequencing technologies to dissect the Ptr genome. The aim is to gain a better understanding of Ptr genome plasticity, to answer how a fungal pathogen like Ptr has evolved to have a mosaic of several effectors in different geographical regions, to explore the role of transposons in genome expansion or compartmentalization, and to define at the genome level, in a meticulous way, the link between virulence evolution and transposons. Ultimately, to release useful genomic resources of well-phenotyped isolates to the research community to be utilized to advance our knowledge on disease emergence.

Ptr has served as a model species for necrotrophic plant pathogens, as it produces various necrotrophic effectors, previously known as host-selective toxins that play essential roles in disease development [2]. To date, three effectors have been identified and are known virulence factors: ToxA is a necrosis-inducing effector, while ToxB and ToxC are chlorosis-inducing effectors [3]. Additional effectors may be involved in pathogenicity and await characterization [4]. ToxA and ToxB are ribosomally synthesized proteins, and while ToxA is encoded by a single-copy *ToxA* gene, ToxB is encoded by multi-copy *ToxB* genes [3]. ToxC is a putative secondary metabolite, and identification of the ToxC-coding gene(s) and its synthesis awaits further characterization [5, 6]. In Ptr, the *ToxA* gene is present only in isolates that secrete the ToxA effector, and no homolog has been identified in non-producing isolates of Ptr [3]. *ToxA* has been found in Ptr and in other related species, whereas *ToxB* has been reported only in Ptr, but its inactive homologs, termed *toxb*, were identified in Ptr and other closely related and distantly related fungal species [7–10].

Ptr isolates are designated into eight different races (races 1 to 8) based on which combinations of these three effectors they secrete [3]. There is an unexplained correlation between Ptr ability to secrete various combinations of effectors and its geographical origins, and in North America and Australia, Ptr isolates are mainly ToxA and ToxC producers, with ToxB producers being extremely rare or absent [4, 11], whereas, in regions encompassing the wheat centre of origin, like the Fertile Crescent and North Africa, all races and effectors are found, even in under-surveyed regions [2, 4], with ToxB producers predominating North Africa [4, 12, 13]. This variation in the presence/absence of effectors suggests a divergent evolution of Ptr and its effectors worldwide.

An independent origin of these effectors was suggested previously [7, 8, 12, 14]. In Ptr, *ToxA* and *ToxB* were never localized to the same chromosome [12]. Yet, *ToxA* was found to reside on a homolog chromosome of essential nature, meaning a chromosome is present in all isolates regardless if the isolate possesses *ToxA* or not. An exception was reported in a race 8 isolate, I-73–1, collected from the Fertile Crescent in Syria. In this isolate, *ToxA* was present on a non-homolog and larger chromosome, which indicates a possible translocation of *ToxA* in the Ptr species [12]. The ability of *ToxA* to transfer horizontally between species and into Ptr has been demonstrated before, and in addition to Ptr, *ToxA* was found in two related species, *Parastagonospora nodorum* and *Bipolaris sorokiniana* [7, 14]. *ToxA* was horizontally transferred via a 14-kb type II DNA transposon named ToxhAT, and multiple horizontal-gene-transfer events including regions surrounding the ToxhAT were likely involved in these species [14]. ToxhAT appears to still be active in *B. sorokiniana* but has likely decayed in *P. nodorum* and may no longer be functional [14]. Unlike the horizontal transfer of *ToxA*, the *ToxB* gene is believed to be vertically inherited from ancestral species. This was indicated by the presence of *ToxB* homologs or *ToxB*-like genes in multiple fungal orders (Ploesporales, Dothideomycetes, and Sordariomycetes) [15]. In this study, we performed a detailed analysis to dissect the regions surrounding *ToxA* and *ToxB* in Ptr.

In addition to the mobility of the effector genes and the surrounding chromosomal segments, the Ptr genome as a whole has been described as highly plastic, with evidence of major chromosomal rearrangements, including insertions, deletions, inversions, and translocations [12, 14, 16–18]. The first full Ptr genome was released by the Broad Institute in 2007, based on a race 1 isolate (Pt-1C-BFP, abbreviated throughout as BFP) from the USA, and was assembled to the chromosome level with the aid of optical mapping [16]. In addition, the full genomes of 11 Ptr isolates from North America and Australia have also been sequenced and are now publicly available [16, 17, 19]. Of those, three (M4, V1, and DW5) were assembled from long-read sequences. The role of DNA transposons and long terminal repeat (LTR) retrotransposons in genome-wide segmental duplications, insertions, and chromosomal fusions in Ptr was investigated [15]. However, genomic regions with AT-rich content, which are associated with repeat-induced point mutations (RIP), were not reported at high frequencies in previously sequenced Ptr isolates from Australia and the USA [17]. RIP is often associated with the “two-speed genome” model in a number of fungal pathogens; this model describes a pattern of physical genome compartmentation with repeat-rich gene-sparse regions and repeat-sparse gene-dense regions with different evolutionary rates [20–22]. Here, we sequenced 40 Ptr isolates of diverse origins and races and performed a whole pangenome analysis with the addition of the reference isolate (BFP), for a total of 41 isolates. These isolates represent all known races including a newly identified atypical race that induces necrosis but lacks the *ToxA* gene [4]. In addition to the pangenome, we provide a detailed look at gene contents, phylogenetic relatedness, and effector gene movement. Furthermore, the polished long-read assemblies representing the full genome of two isolates (I-73–1 a race 8 isolate from Syria and produces all three effectors, and therefore known as the most complex race so far in Ptr, and D308, a Canadian race 3 isolate that produces only the ToxC, and carries inactive homolog of *ToxB*) were explored. The structural organization and genome compartmentation in these two isolates were analysed and in comparison with three previously published genomes (BFP and DW-5 from USA and M4 from Australia).

## Results

### Hybrid assemblies capture a better estimate for genome size

The de novo assemblies of Illumina short-reads indicated a variable genome size ranging from 34.1 to 36.9 Mb, with an average of 34.82 Mb. The smallest assembly was for isolate T128-1 (34.12 Mb), which has been identified as an atypical isolate with a novel virulence phenotype [4]. The largest assemblies were for isolates 92-171R5 (36.8 Mb; race 5) and G9-4 (36.97 Mb; race 4).

The hybrid assembly (PacBio RS II and Illumina HiSeq data) of isolate I-73–1 contained 39 contiguous sequences, with 11 chromosome-sized contigs greater than 2 Mb. Similar results were obtained with isolate D308, whose assembly contained 70 contiguous sequences, and 11 contigs greater than 1 Mb with an additional two smaller contigs representing the majority of the genome. Genome completeness was assessed by Benchmarking Universal Single-Copy Orthologs (BUSCO) using a set of conserved fungal genes, and all isolates (except G9-4) were assessed as > 99% complete. The short-read assemblies of I-73–1 and D308 were ~ 5.3 Mb smaller than the long-read-based assemblies. Summary statistics for all assemblies are available in Table 1.

**Table 1:** Summary statistics for 40 isolates of *Pyrenophora tritici-repentis* sequenced with Illumina HiSeq X and assembled with Shovill/SPAdes, and two isolates sequenced with PacBio and assembled with Flye/Pilon (table is sorted by race then isolate name)

Isolate	Race	NE	Year	Location^a^	Host	Size (MB)	Contigs	GC	TE	N50	Genes	BUSCO	Accessory
								**(%)**	**(%)**	**(kbp)**		**(%)**	**(%)**
ASC1	1	AC	1985	Manitoba	*T. aestivum*	34.78	6495	50.9	6.9	65.5	13,124	99.3	22.5
I-33–1	1	AC	2001	Azerbaijan	*T. aestivum*	35.06	6850	50.9	6.9	75.2	13,055	99.5	22.1
L3-1	1	AC	2016	Alberta	*T. durum*	34.93	6666	50.9	6.9	77.2	13,115	99.4	22.4
L4-1	1	AC	2016	Alberta	*T. durum*	34.74	6361	50.9	6.9	72.4	13,063	99.6	22.1
SW20-7	1	AC	2016	Saskatchewan	*T. durum*	35	6568	50.9	7.2	76.2	13,116	99.4	22.5
SW2-1	1	AC	2016	Saskatchewan	*T. durum*	35.11	6789	50.9	6.9	74.3	13,116	99.7	21.3
SW21-1	1	AC	2016	Saskatchewan	*T. durum*	34.62	6282	50.9	6.8	74.3	12,965	99.4	21.6
SW21-7	1	AC	2016	Saskatchewan	*T. durum*	34.97	6741	50.9	7	74.7	13,126	99.4	22.5
SW21-8	1	AC	2016	Saskatchewan	*T. durum*	34.96	6631	50.9	6.9	75	13,073	99.4	22.2
SW7-5	1	AC	2016	Saskatchewan	*T. durum*	35.65	6701	50.8	6.9	73.7	13,490	99.6	24.6
86–124	2	A	1986	Manitoba	*T. aestivum*	34.9	6832	50.9	6.9	60.7	13,209	99.2	23
AB88-2	2	A	2010	Alberta	*T. aestivum*	34.83	6465	50.9	6.9	75.3	13,126	99.5	22.5
L2-1	2	A	2016	Alberta	*T. durum*	34.96	6923	50.9	7.1	72.9	13,130	99.4	22.5
SW1-2	2	A	2016	Saskatchewan	*T. durum*	35.08	6599	50.9	7.1	71.2	13,365	99.4	24
SW15-1	2	A	2016	Saskatchewan	*T. durum*	34.83	6272	50.9	6.6	78.8	13,201	99.7	22.9
T132-2	2	A	2017	Tunisia	*T. durum*	34.41	6472	50.9	6.8	57.1	12,935	99.4	21.3
331–2	3	C	1985	Manitoba	*Triticum* sp.	34.44	6828	51	6.9	55.6	12,909	99.5	21.2
D308	3	C	1985	Manitoba	*T. aestivum*	34.33	6809	51	6.8	58.5	12,826	99.1	N/A^c^
D308^b^	3	C	1985	Manitoba	*T. aestivum*	39.6	70	50.8	18.6	3667	12,501	99.6	18.7
I-72–1	3	C	2001	Syria	*Triticum* sp.	34.35	6734	51	6.8	58.6	13,011	99.5	21.8
I-72–7	3	C	2001	Syria	*Triticum* sp.	34.35	6696	51	7	58.9	12,901	99.4	21.2
SC29-1	3	C	1999	Saskatchewan	*T. durum*	34.19	6464	51	6.8	59	12,951	99.5	21.5
SW21-5	3	C	2016	Saskatchewan	*T. durum*	34.66	6619	51	7.2	63.5	13,029	99.5	22
90–2	4	Absent	1990	Canada	*T. aestivum*	35.22	3818	50.8	7.2	225.9	12,909	99.5	21.3
G9-4	4	Absent	2016	Alberta	wild grass	36.97	8035	50.7	9.8	78.2	13,148	98.7	23
T126-1	4	Absent	2017	Tunisia	*T. durum*	34.15	6373	51	6.5	62.4	12,837	99.6	20.6
92-171R5	5	B	1992	Saskatchewan	*T. aestivum*	36.81	14,647	51.1	10.2	45.1	13,393	99.3	24.1
Alg3-24	5	B	1993	Algeria	*T. durum*	34.3	6098	50.9	6.6	73.6	12,900	99.6	21.1
Alg4x-1	5	B	1993	Algeria	*T. durum*	35.71	8072	50.9	7.5	71	13,193	99.4	22.9
I-17–2	5	B	2001	Azerbaijan	*T. durum*	34.25	6555	51	7	62.5	12,820	99.6	20.7
I-34–5	5	B	2001	Azerbaijan	*Triticum* sp.	34.29	6315	51	6.8	61.9	12,841	99.6	20.8
I-35–56	5	B	2001	Azerbaijan	*T. durum*	34.24	6516	51	6.9	62.6	12,918	99.5	21.3
I-36–1	5	B	2001	Azerbaijan	*T. durum*	34.36	6467	50.9	6.7	62.4	12,881	99.5	21
AlgH1	6	BC	1993	Algeria	*T. durum*	34.74	6902	51	6.9	61.7	13,159	99.3	22.7
AZ35-5	7	AB	2001	Azerbaijan	*T. durum*	35.3	7165	50.9	7.1	72.1	13,239	99.5	23.2
T176-2	7	AB	2017	Tunisia	*T. aestivum*	34.7	6896	50.9	7.3	57.1	13,141	99.5	22.6
T181-1	7	AB	2017	Tunisia	*T. durum*	34.78	6718	51.2	6.5	57.5	13,583	99.4	25.1
I-34–1	8	ABC	2001	Azerbaijan	*T. durum*	34.38	6405	50.9	6.6	64.5	13,036	99.5	22
I-35–18	8	ABC	2001	Azerbaijan	*T. durum*	34.85	6698	50.9	7	65.3	13,071	99.4	22.9
I-73–1	8	ABC	2001	Syria	*T. aestivum*	34.62	6619	50.9	6.8	63.4	12,941	99.5	N/A^c^
I-73–1^b^	8	ABC	2001	Syria	*T. aestivum*	39.9	39	50.8	18.3	3647	12,744	99.6	20
T128-1	Atypical	B	2017	Tunisia	*T. durum*	34.12	6095	51	6.4	59.1	13,002	99.6	21.7

*NE* Necrotrophic effector

^a^Alberta, Saskatchewan, and Manitoba are provinces within Canada

^b^Long-read assemblies

^c^Not included in pangenome

### Ptr has an open pangenome with high accessory gene content and unique genes for non-pathogenic and weakly virulent isolates

**Fig. 1:**
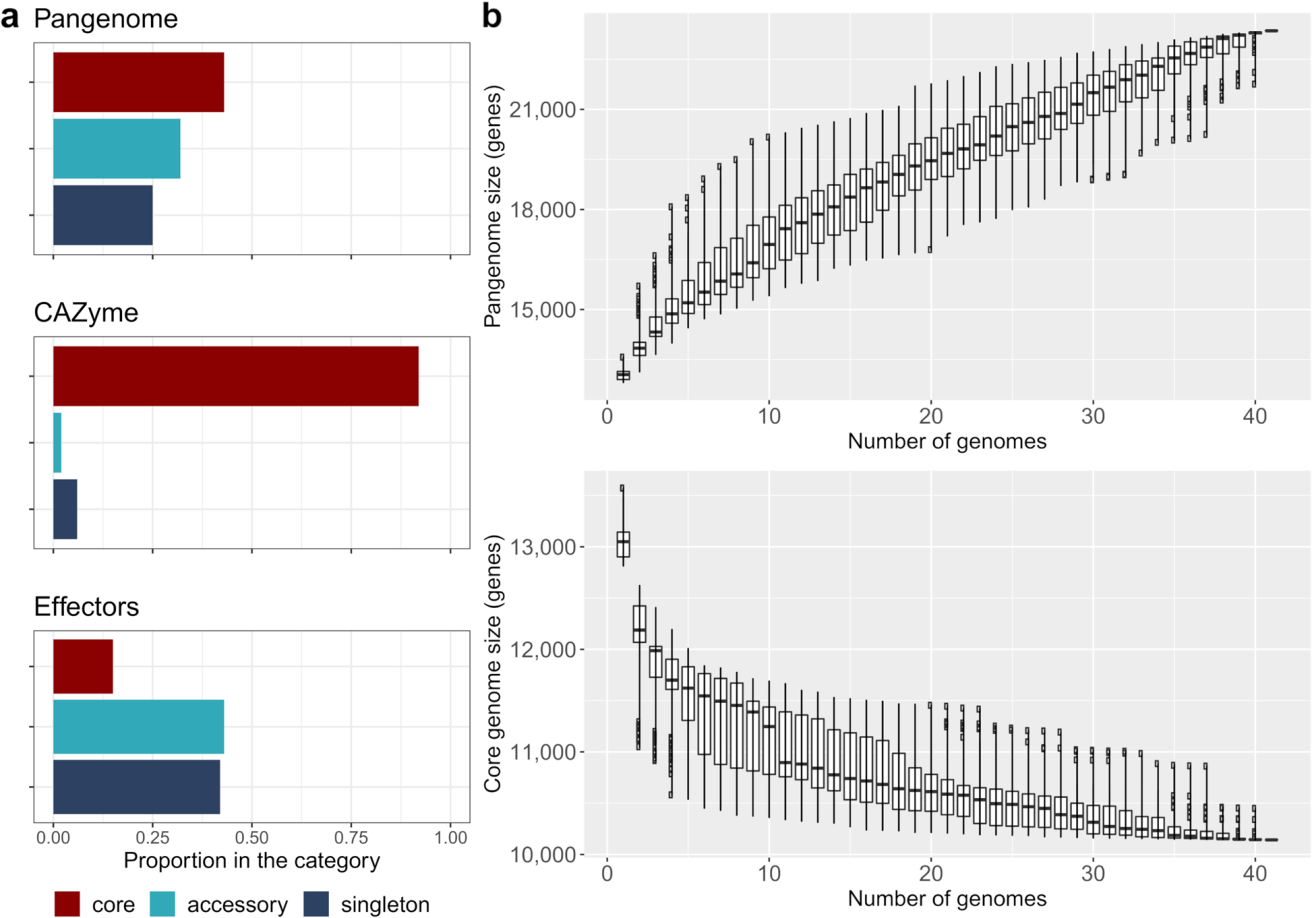
The pangenome of *Pyrenophora tritici-repentis*. **a** Proportion of genes present in the core, accessory (excluding singletons), and singleton sets for pangenome, CAZymes, and effectors. **b** Total number of unique genes and core genes within the constructed pangenome of *Pyrenophora tritici-repentis* as genomes are added. Starting genome was always Pt-1C-BFP, with subsequent genomes added randomly, iterated × 100, and visualized as boxplots

**Fig. 2:**
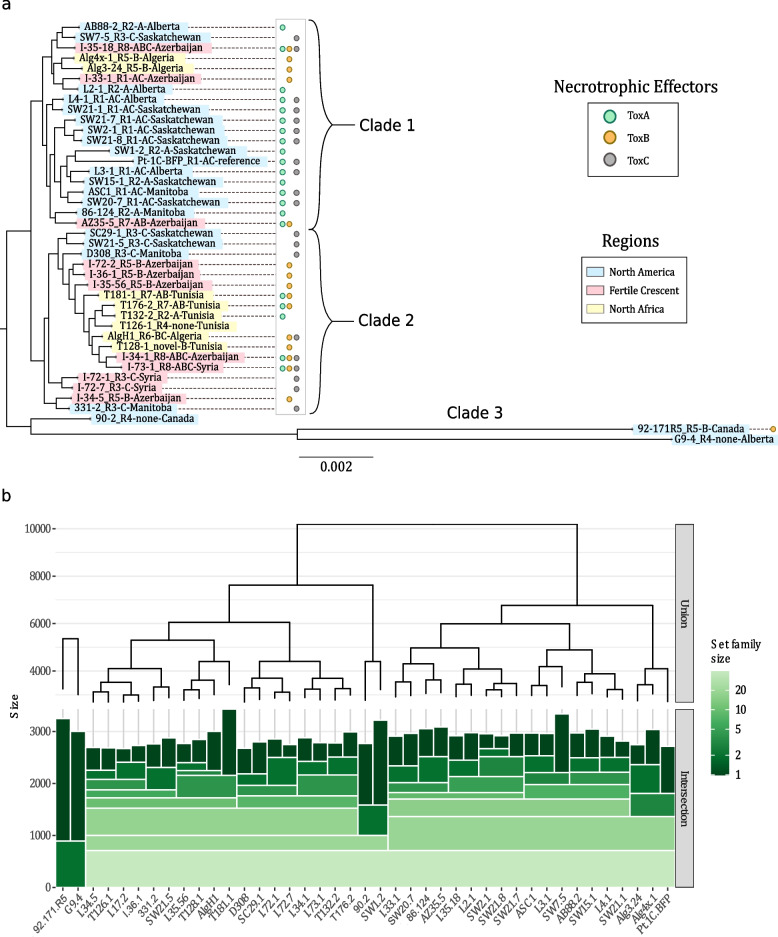
Evolutionary relationships of isolates from the constructed pangenome of *Pyrenophora tritici-repentis*. **a** Maximum-likelihood phylogenetic tree of *Pyrenophora tritici-repentis* based on aligned and concatenated proteins present in the core genome (total 10,159 genes). Isolate names are followed by their race (R1 to R8) designation, necrotrophic effector(s) produced (A, B, and/or C), and their location of origin (isolates collected within Canada show province names; isolates from outside Canada show country of origin). Presence of *ToxA* (green circles) and *ToxB* (orange circles), confirmed via BLAST, and presence of *ToxC* (grey circles) determined by phenotyping. **b** Hierarchical sets of *Pyrenophora tritici-repentis* accessory genes, set family size represents the number of genes shared by isolates in each group, where inclusion in a group is based on any given isolate overlapping with the horizontal bars

On average, each isolate contained 13,071 predicted genes, and collectively, 522,848 predicted genes across all isolates were reported. After grouping by amino acid sequence similarity with a similarity threshold of ≥ 90% (E10^−4^) and local synteny (conserved gene neighbourhood scores) via Pangloss [23], we were left with 23,454 groups of unique homologous genes. It must be noted that Pangloss will not group more than one gene per isolate into a gene cluster, and therefore, the following statistics may include genes with multiple copies. In total, 10,159 genes were identified as core (conserved across all isolates) (43% of total pangenome) (Fig. 1A). The remaining 13,295 gene models (~ 57% of the total pangenome) were the accessory genome, with individual isolates carrying 2661 to 3424 accessory genes. Within the accessory genome were 7330 singletons (genes present in only one isolate) (55% of the accessory genome; 31% of the total pangenome) which were parsed via a custom script. Within the singletons, a large number 3293 (44%) were present only in isolates from the outgroup of the phylogeny (Fig. 2A), including 90–2 (435 genes), 92-171R5 (1598 genes), and G9-4 (1204 genes). Additionally, isolate T128-1, which exhibits a novel necrotic phenotype [4], contained 54 unique genes. Post-Pangloss BLAST searches of singletons suggest a small number of singletons (~ 6%) are present in multiple copies (90% identity over 80% of query length) which suggests the number of singletons is over-estimated.

Genes were binned into sets based on the presence or absence of genes to provide insights into gene gains and losses in Ptr (Additional file 1). Each set is indicative of how many isolates share a particular gene (e.g. set 2 shows genes shared by two isolates, set 3 by three isolates, etc.). Sets 1 and 41 were not included, as these represent the singleton and core gene set, respectively. The result of this sub-setting is groups of genes (based on how many isolates contain the gene) many of which are likely gene gains and losses throughout Ptr.

Isolates carrying the *ToxA* gene had significantly larger accessory genomes (mean of 2987 genes) compared with isolates lacking *ToxA* (mean of 2820 genes) based on a two-sample *t*-test (*p* = 0.002). No such relationship was found with *ToxB* or putative *ToxC-*carrying isolates. An ANOVA followed by Tukey’s HSD test indicated that race 7 (*ToxA*, *ToxB*) and race 3 (*ToxC*) isolates also had significantly different accessory genome sizes (average of 3162 and 2779 genes, respectively; *p* = 0.04), with no other notable differences between races. Additionally, the total number of genes present in the pangenome increased steadily with each additional genome (Fig. 1B), and the number of core genes appears to have reached a stable level after the addition of 38 genomes (Fig. 1B). The continual increase of genes per genome and the large accessory genome (57%) are strong indications that Ptr has an open pangenome. It is worth noting as well, that when the three outlier isolates (90–2, 92-171R5, and G9-4) are removed from the analysis, the genome curve is similar (not shown) and still indicates Ptr possesses an open pangenome.

### Ptr core protein and SNP phylogenies exhibit clustering based on necrotrophic effector secretion

A maximum-likelihood tree to estimate genetic relatedness among Ptr isolates was constructed using an alignment of the 10,159 core proteins and showed that most isolates partitioned into two major clades supported by monophyletic branches (Fig. 2A). Clade 1 contained 20 isolates, most of which were ToxA-producing Canadian isolates classified as races 1 and 2. Few isolates in clade 1 were ToxB producers; those that were ToxB producers were from Azerbaijan and Algeria. Clade 2 contained 19 isolates, most of which were ToxA non-producing races 3 and 5, which mostly originated in the Fertile Crescent and nearby regions. The Canadian isolates in clade 2 were all classified as race 3 (ToxC producers). Three isolates (92-171R5, 90–2, and G9-4) clustered as an outgroup defining clade 3. 92-171R5 is the only race 5 isolate identified in Canada and was described as weakly virulent [24]. Isolates 90–2 and G9-4 were classified as non-pathogenic race 4 [13, 25].

Heirarchical set groupings based on accessory proteins revealed a similar structure as the core protein phylogeny (Fig. 2B), with the only exceptions being in which major clades the isolates 90–2 (race 4) and SW1-2 (race 1) were placed. These two isolates shared a high number of genes not present in other isolates, defining their placement within the hierarchical set. Clusters in the hierarchical sets (horizontal bars) were defined by the presence or absence of accessory genes and do not take into account any sequence variation in the homologs used to construct the core protein phylogeny, but highlight that Ptr isolates in this study were mainly distributed in two clades: one is ToxA-producing isolates, with few exceptions, and a second contains mainly ToxA non-producing isolates of races 3 and 5. These race 3 and 5 isolates were mainly isolated from durum (tetraploid) wheat, whereas the ToxA producers were mainly collected from bread wheat (hexaploid). This may reflect on the genetic relatedness accumulated in relation to the host ploidy adaptation. Additional phylogenetic trees based on SNP data also closely match the core protein phylogeny and the hierarchical set trees in terms of branching, with the divergent isolates separating even further (Additional file 2).

### Predicted genes and effectors

Of the 23,454 gene clusters in the Ptr pangenome, only 46% were present in the Pfam functional database. Of the 10,159 core genes, 6951 (69%) had some described functionality (e.g. domain, family, etc.). However, of the 13,295 accessory genes, only 3782 (29%) were annotated with a functional domain, the remaining being hypothetical proteins. Core gene protein lengths were significantly greater than accessory proteins, with mean lengths of 482 aa and 241 aa, respectively, and medians of 411 aa and 156 aa. Singleton proteins were marginally smaller than other accessory proteins, with a mean length of 232 aa (median 156 aa). A small number of proteins [26] were quite large, with lengths exceeding 2000 aa, and six of those exceeding 5000 aa; the largest protein was 9750 aa in length. Many of these genes were annotated in previously published Ptr genomes.

Effectors play a major role in establishing infection in necrotrophic pathogens. In total, 729 potential effectors (3.1% of the total pangenome) were predicted in Ptr. Of these, 106 (14.5%) were present as core genes, and 626 (85%) were accessory genes, with 313 of the accessories being singletons (Fig. 1A). Omission of the non-pathogenic race 4 isolates (90–2, G9-4, and T126-1) revealed 584 unique effectors, with 127 genes in the core and 457 as accessory (198 singletons). Nearly half of the potential effectors were singletons present in the weakly virulent isolate 92-171R5.

### CAZymes are highly conserved in Ptr

We identified 305 unique CAZymes from 91 CAZyme families that are known to be active on cell wall polysaccharides. Of these, 281 (92%) were observed in all isolates; the number of proteins within the CAZyme families was predominantly conserved across all isolates (Fig. 1A). Although numbers were similar between isolates and were related at the sequence level, there may be functional differences that remain to be explored. CAZyme families associated with plant cell wall degradation and fungal phytopathogenesis were abundant (Additional file 3). These included GH5 cellulases and endoglucanases, GH43 arabinases and xylosidases, AA9 lytic cellulose monooxygenases, CE1 hemicellulases, and CE5 cutinases [27–32]. Protein sequences within these families accounted for 31% of CAZymes identified. It should be noted that not all CAZymes are associated with pathogenesis, and many are likely involved in routine cellular activities, such as fungal cell wall remodelling and glycoprotein maturation.

### Extensive chromosomal structural organization in the Ptr genome

**Fig. 3:**
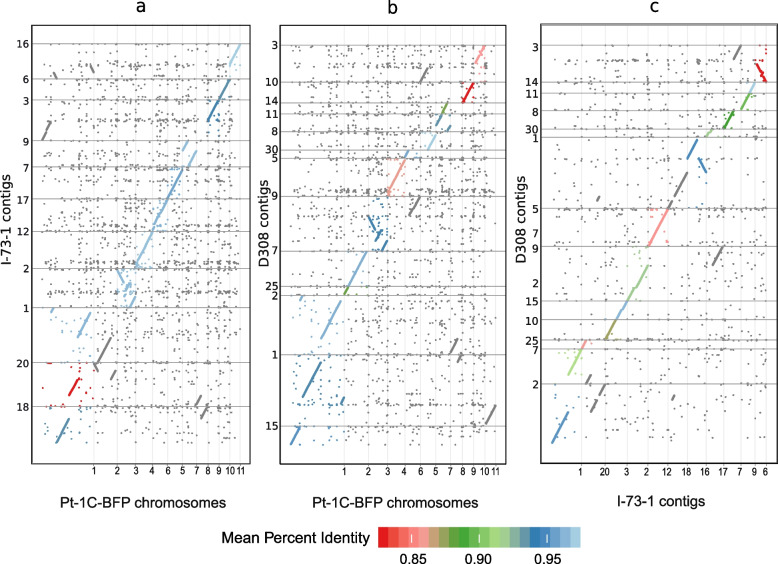
Full genome alignments of newly sequenced long-read assemblies of *Pyrenophora tritici-repentis* isolates I-73–1 (race 8) and D308 (race 3) to the reference isolate Pt-1C-BFP (race 1). **a** Pt-1C-BFP and I-73–1; **b** Pt-1C-BFP and D308; **c** I-73-1 and D308

**Fig. 4:**
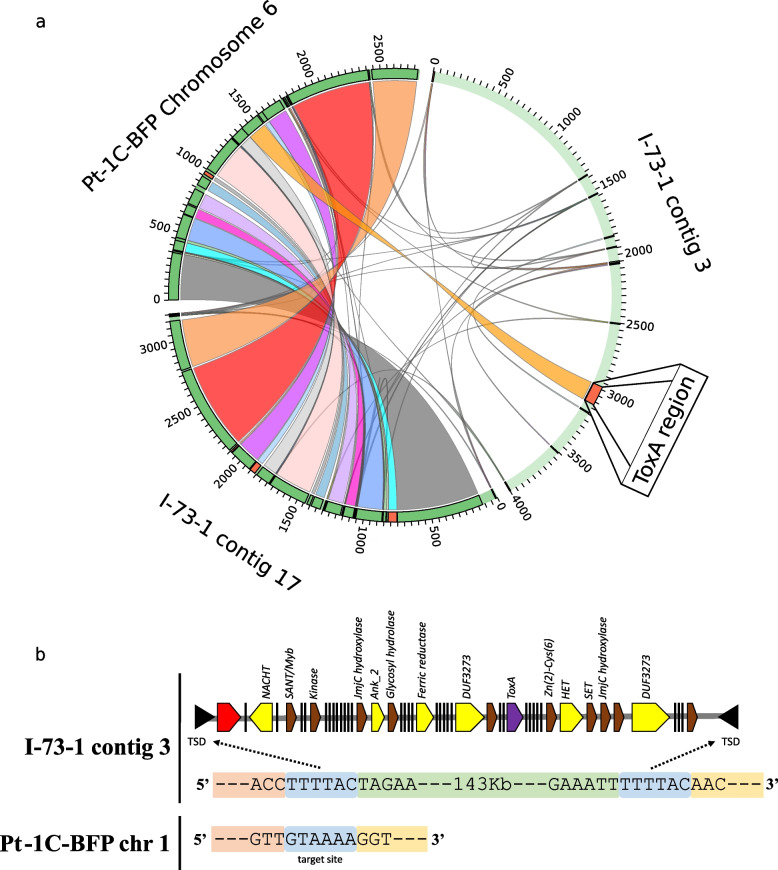
Evidence of a likely translocation of the *ToxA* containing region between two races of *Pyrenophora tritici-repentis* facilitated Starship transposon ‘Horizon’. **a** The *ToxA* containing chromosome 6 of isolate Pt-1C-BFP (race 1) is compared with the *ToxA* containing contig 3 and contig 17 of isolate I-73–1 (race 8). Pt-1C-BFP chromosome 6 aligns almost completely to I-73–1 contig 17, with the exception of the *ToxA* containing region, which aligns to contig 3. The majority of contig 3 aligns to either Pt-1C-BFP chromosome 1 or chromosome 9 (Fig. 3). **b** Schematic of the ‘Horizon’ Starship (red gene = tyrosine recombinase; yellow = known Starship cargo genes; purple = *ToxA* gene; brown = annotated gene; black bar = gene with no known function)

Full genome alignment between the hybrid assemblies of isolate I-73–1 (race 8) and the reference BFP (race 1) showed 11 contigs in I-73–1 of comparable size and content to the 11 chromosomes of the reference BFP. Alignment of D308 (race 3) indicated 10 contigs of comparable size, with an additional three smaller contigs completing a full alignment to BFP. BFP chromosomes 4 and 7 appear to be co-linear between all three isolates (contigs 12 and 9 in I-73–1 and contigs 5, 11, and 14 in D308) (Fig. 3). Chromosomes 3, 5, 6, and 10 in BFP were also mostly homologous with contigs 2, 7, 17, and 6, respectively, in I-73–1 (Fig. 3); the only notable exception was the *ToxA* containing region detailed further below (Fig. 4A).

Large-scale chromosomal rearrangements such as chromosome fusions and inversions were observed between I-73–1 and BFP. This was illustrated clearly by the chr 1 fragmentation in I-73–1, whereas chr 1 is the largest chromosome in BFP (10.2 Mb). A significant section of chr 1 aligned with contig 18 in I-73–1, but the remaining sections were found to be distributed between four contigs: 1, 3, 16, and 20. Additionally, BFP chr 8 was split between I-73–1 contigs 18 and 20. Approximately 30% of chr 3 (contig 2 in I-73–1) was inverted. Similar rearrangements on a smaller scale were observable in the alignment dotplot (Fig. 3).

Similar to I-73–1, a chr 1 fragmentation was observed in D308. However, the fragmentation in the latter was less severe, with sections aligning to three contigs (D308 contigs 1, 2, and 15). There was a collinearity between D308 contigs 11 and 14 and chr 7 in BFP, and hence, these two contigs likely represent a single chromosome that would align fully with BFP chromosome 7 and I-73–1 contig 9. Furthermore, D308 contigs 7 and 25 fully aligned with BFP chr 2. The genome architectures of D308 and I-73–1 appear to exhibit higher collinearity between each other than to isolate BFP (Fig. 3). We found similar types of rearrangements in alignments with M4 and DW-5 although they were fewer in number (not shown). There was no evidence for the presence of supernumerary chromosomes in I-73–1 or D308.

### ToxA is present on a Starship class transposon and ToxB is present within a massive putative transposon

*ToxA* in BFP was located on chr 6 (2.8 Mb), but in I-73–1, *ToxA* was carried on contig 3 (4.0 Mb) which aligns with BFP chr 1 and 9. While BFP chr 6 was present in I-73–1 as contig 17, the contig lacked *ToxA* and a 143-kb segment (Fig. 4A). This validates the previously reported translocation of *ToxA* in I-73–1 [12]. Edge analysis and review of gene content within the 143-kb segment indicated target site duplications (or short direct repeats) at the edges and a tyrosine recombinase (gene_09853; DUF3435 domain) at the 5′ end, initially suggesting this element is a *crypton* [33]. Recently, however, a new class of large mobile elements called *Starships* have been defined [34]. An examination of other genes present within the transposon suggests that this element is also a *Starship* with many similarities, including the DUF3435 tyrosine recombinase ‘captain’, DUF3273 domains, ferric reductase, ankyrin repeats, heterokaryon incompatibility, NACHT nucleoside triphosphatase, and target site duplications (Fig. 4B) [34]. A full list of genes present is available in Additional file 4. The target site itself was identifiable in BFP chr1 (Fig. 4B), M4 (not shown), and D308 (not shown). We have named this new *Starship* ‘Horizon’. Additionally, an alignment of the ToxhAT transposon, which facilitated the movement of *ToxA* between Ptr, *Parastagonospora nodorum*, and *Bipolaris sorokiniana* [14], showed that this smaller 14-kb transposon is nested within Horizon.

**Fig. 5:**
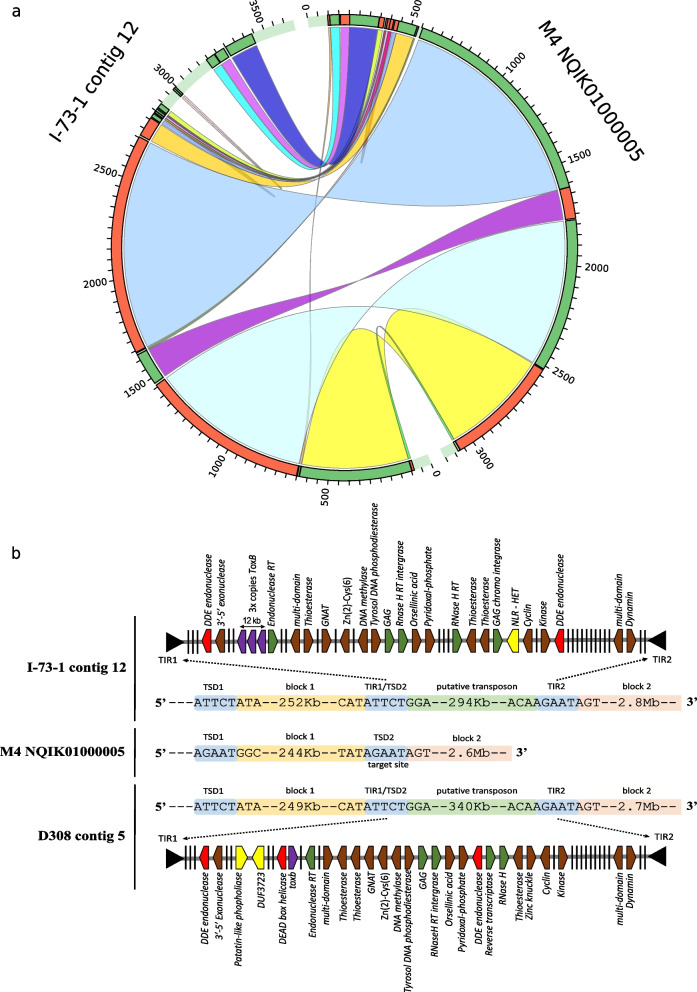
Evidence of putative Starship transposon ‘Icarus’ associated with *ToxB* in *Pyrenophora tritici-repentis* **a** Circular alignment of contig 12 from race 8 isolates I-73–1 and contig NQIK01000005 from race 1 isolates M4. A large 294-kb region which contains three copies of the *ToxB* (black arrow) is visible, and this section did not align with any other contig of M4. The edges of the 294-kb region revealed terminal inverted repeats suggesting transposon activity. **b** Schematic of ‘Icarus’ Starship (red genes = associated with type II DNA transposons; green = associated with type I RNA transposons; yellow = known Starship cargo genes; purple = *ToxB/toxb*; brown = annotated gene; black bar = gene with no known function)

Four copies of *ToxB* were found in the I-73–1 long-read assembly; each copy contained a single 49-bp exon. Three copies were located within a 12-kb region on contig 12 (homologous to chr 4 in BFP), and the fourth copy was present on a small 6-kb contig (contig 13) which failed to assemble with the chromosome-sized contigs. The Australian isolate M4 (race 1) offered a better scaffolding quality than BFP to examine the 12-kb *ToxB* region, and the alignment of I-73–1 contig 12 to M4 contig NQIK01000005 indicated an even larger 294-kb gap around the region of *ToxB* (Fig. 5A). Examination of the edges of this gap revealed the presence of terminal inverted repeats (TIRS) in I-73–1 and a potential target site duplication in M4 (Fig. 5B). This same 294-kb transposon with the same TIRs is present in D308 on contig 9, and although the *ToxB* gene is missing in D308, its inactive homolog *toxb* is present within the same transposon as a single copy (Fig. 5B and Additional file 5A). While the DUF3435 tyrosine recombinase ‘captain’ was absent in both I-73–1 and D308, the heterokaryon incompatibility gene was present in I-73–1 while DUF3273 and patatlin-like phospholipase genes were present in D308. Additionally, DDE endonucleases (D:aspartic acid; E:glutamic acid) commonly present in class II DNA transposases were present in both isolates along with many other genes associated with class I RNA intermediate transposons (Fig. 5B). These findings taken together suggest this region may be either disabled or a type of Starship which makes use of something other than a tyrosine recombinase. We have called this putative Starship ‘Icarus’.

*ToxB/toxb* genes were found here to be located on an essential chromosome/contig in Ptr, as the same chromosome/contig is present in other isolates that do not carry the *ToxB* gene (BFP and M4). DW5 was reported to carry 10 copies of *ToxB* [35]. Our BLAST output indicated 10 copies, but eight of these were on contigs ≤ 126 kb, which may indicate misassembly. Only two copies of *ToxB* were on contigs of comparable size to chromosomes (CM025819 at 3.4 Mb and CM025824 at 2.2 Mb, respectively). These contigs aligned with BFP chr 5 and 11 and I-73–1 contigs 7 and 16, respectively (Additional file 5B). The locations of *ToxB* copies in DW-5 do not appear to have co-linearity to the putative transposon in I-73–1 and D308, which may indicate additional transposon activity associated with *ToxB*.

The chromosomes and contigs that possess *ToxB*/*toxb* were fully (or nearly fully) present in all isolates examined, indicating they are essential in nature or contain essential genes for Ptr. Additionally, the presence of core and accessory genes and their ratios in isolates I-73–1 and D308 were examined, and for I-73–1 contig 12, the ratio of core to accessory genes was 3.4, while for D308 contig 9 the ratio was 3.8, supporting the essential nature of these chromosomes (Additional file 6).

### Ptr exhibits a ‘one-compartment’ genome

**Fig. 6:**
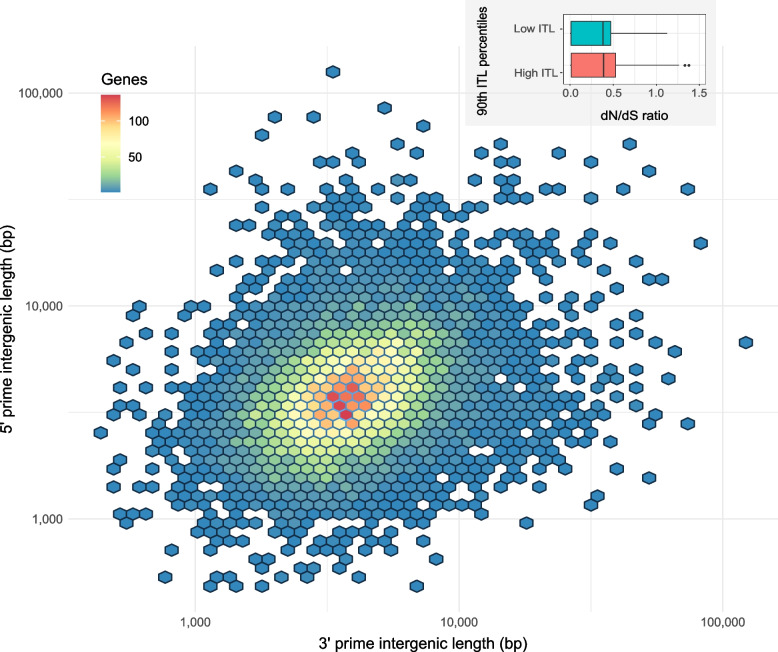
Intergenic distances of all genes in *Pyrenophora tritici-repentis* isolate I-73–1. The 3′ intergenic length (*x*-axis) is the distance (bp) from the 3′ end of the current gene to the 5′ end of next, and the 5′ intergenic length (*y*-axis) is the distance from the 3′ end of the previous gene to the 5′ end of the current gene. Inset (top right) shows dN/dS ratios for genes from the 90th ITL percentiles (i.e. genes furthest from others and genes nearest to others; outliers omitted for clarity)

The genome of many plant pathogens exhibits a two-compartment arrangement, one with AT-rich gene-sparse regions (GSR) where effectors and TEs are embedded, and a second with gene-dense GC-rich regions (GDR) [20–22]. In order to evaluate if the Ptr genome exhibited compartmentalization, the intergenic distances (the number of nucleotides between genes) were measured and found to average ~ 4600 bp in both I-73–1 and D308. Density plots of the 5′ and 3′ intergenic distances showed a single hot spot at a similar size range (Fig. 6). A scattering of genes in the upper right portion of the plot indicates that some genes are in gene-sparse regions, with intergenic distances as high as 100,000 bp in certain cases. The single hot spot (rather than two) is indicative of a ‘one-compartment’ genome, and although there are a number of genes in gene-sparse regions, their frequency does not support a ‘two-compartment’ genome. Genes with the highest and lowest intergenic distances (90th percentiles; labelled GSR set and GDR set respectively; Additional file 7) were selected for evolutionary rate analysis by comparison of non-synonymous (dN) and synonymous (dS) substitutions. The I-73–1 GDR set contained 163 genes and the GSR set contained 134 genes, and of these, the majority (> 88% for each set) had dN/dS ratios < 1 indicating negative selection pressure. Comparison of dN/dS means from the GDR and GSR sets revealed no difference in evolutionary rates between the sets (*p* = 0.6207) (Fig. 6). Similar results were found with D308 (Additional file 8). Additionally, analysis with RIPper showed that < 2% of the I-73–1 and D308 genomes were affected by RIP, with < 40 large RIP affected regions (LRARs) present in each.

### Ptr genome and expansion by transposable elements

**Fig. 7:**
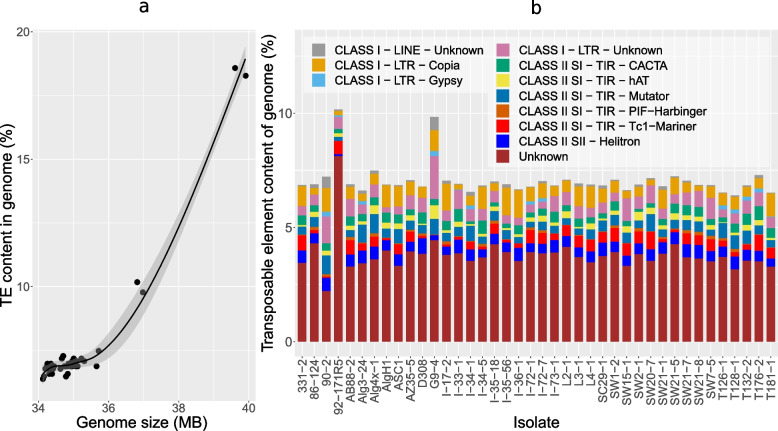
Transposable element content and genome size variation of *Pyrenophora tritici-repentis* from short-read assemblies. **a** Contribution of TEs (%) to total genome size across 40 isolates. **b** Contribution of each TE class to genome size for individual isolates

Transposable element (TE) content in the short-read assemblies ranged from 6.4 to 10.2% of the assembled genomes (Fig. 7A). The highest TE content occurred in isolates 92-171R5 (10.2%) and G9-4 (9.8%). A large proportion of the TEs in 92-171R5 were classified as ‘unknown’ repetitive elements. In G9-4, however, the increased number of TEs was primarily class I-LTRs. The high TE content in these two isolates also correlates with larger genome sizes relative to the other assemblies (Fig. 7B). TE content in 12 isolates (excluding 92-171R5 and G9-4) exceeded 7%, and the remaining 26 isolates have TE content ranging between 6.4 and 7.0%. All assemblies contained significant numbers of ‘unknown’ repetitive elements. The program EDTA (Extensive de-novo TE Annotator) uses structural features to identify intact TEs at the beginning and then classifies them into families based on coding features. RepeatModeler2 was used to identify repeats not initially reported, but due to a lack of homology and coding features, they were labelled as ‘unknown’ TEs.

The long-read assemblies of isolates I-73–1 and D308 showed a significantly larger number of transposable elements (> 150%) when compared to short-read assemblies of the same isolates. TEs represented 6.8% and > 18.3% for the short- and long-read assemblies, respectively. In both isolates, the increased TE content was due to a greater incidence of class I transposons, primarily Copia and Gypsy elements. Additionally, many previously uncategorized transposons (labelled as ‘unknown’ in the short reads) were reclassified with accurate transposon labels. Almost all groups of transposons identified in the short-read assemblies showed some increased incidence in the long-read assemblies, with the exceptions of the Tc1-Mariner, Helitrons, and PIF-Harbinger classes, which were consistent between assembly types.

## Discussion

In this study, we performed a global pangenome analysis of Ptr, an important foliar pathogen of wheat. In total, 40 newly sequenced genomes were added and 41 carrying various combinations of all known and unknown effectors were analysed. These isolates represent various geographical origins, extending from regions where wheat is of relatively recent introduction to regions encompassing the centre of wheat origin. We identified major rearrangements at the chromosomal level among five assembled Ptr genomes, two of which were generated in this study (I-73–1 and D308) and three of which were previously described (BFP, M4, and DW5). These rearrangements included chromosomal fusions, segment inversions, and translocations. We showed that the Ptr genome appears to tolerate large-scale structural reorganizations, genome size variation, and has remarkable plasticity. Previously, a worldwide collection of Ptr isolates, some of which were included in this study, had shown the independent chromosomal location of the virulence genes *ToxA* and *ToxB* and extensive genome plasticity (karyotypes), where aneuploidy, proliferation of repetitive DNA, and transposon activities were suggested as driving mechanisms [12]. Here, we showed plasticity in the Ptr genome is likely to be facilitated by the proliferation of TEs and the expansion of the accessory genome, in addition to the role of transposable elements in virulence gene shuffling.

Fungi are known to display high variability in their genomes, and the ability of plant pathogenic species to gain virulence genes embedded in a ‘pathogenicity island’ and carried on supernumerary chromosomes that transfer horizontally as whole or in part is well described [36]. Recently, more reports have emerged on the ability of virulence genes to transfer on large transposons among plant pathogens [34]. Overall, the ability of pathogenic fungi to evolve rapidly to invade their hosts or cause outbreaks is illustrated through their ability to recombine, mutate, and shuffle their genetic components either vertically or horizontally. Traditionally, a ‘two-compartment’ genome has been associated with a ‘two-speed’ genome in pathogenic fungi. In this case, Ptr appears to possess a ‘one-compartment’ genome; however, this does not preclude the species from having a ‘two-speed’ genome driven by copy-number variation and TEs not associated with compartmentalization [21], as we know *ToxB* is present as multiple copies in Ptr. Other effectors or genes associated with virulence may be present in multiple copies, but this remains to be explored.

### The mobility of ToxA in Ptr is facilitated by the Starship transposon ‘Horizon’

*ToxA* is present in other fungal species such as *Parastagonospora nodorum* and *Bipolaris sorokiniana* [7, 14]. The independent horizontal transfers between these species have been explored in detail, with the transfer being facilitated via a hAT transposon dubbed ToxhAT, which is 14 kb in size [14]. Previously, *ToxA* was found to be located on the same essential chromosome in all tested Ptr isolates with the exception of I-73–1, where it was shown to be translocated to a larger non-homologous chromosome, as indicated by pulse-field gel electrophoresis followed by southern hybridization [12]. Although ToxhAT is present, we validate the location of *ToxA* in I-73–1 within ToxhAT, but also nested on a much larger putative 143-kb *Starship* transposon, which we have named ‘Horizon’. This mobile element was integrated into an essential chromosome (contig 3; 4 Mb) in I-73–1, corresponding to chr 9 in BFP. The *ToxA* coding gene in all Ptr isolates tested here has a very conserved sequence belonging to one haplotype (PtrH1) [37] (data not shown), and this supports the hypothesis of its recent integration into the Ptr species [7, 38]. This new class of transposable elements, *Starships*, are extremely large cargo-carrying elements associated with the movement of diverse gene sets such as biosynthetic clusters, virulence factors (e.g. *ToxA*, *Ago1*, and *OCH1*) [39, 40], heavy metal resistances, and metalloreductases. *Starships* were identified in a broad set of Ascomycete fungi sharing a common ancestor approximately 400 million years ago. There is also evidence for the involvement of *Starships* in at least two horizontal-gene transfer events [34].

The movement of *ToxA* in different types of mobile elements in Ptr and the nesting of transposons is indicative of this pathogen’s ability to evolve and acquire virulence rapidly. The nesting of mobile elements with virulence factors has been linked to the evolution of pathogenicity in other species as well [14, 41, 42] and the association of TEs with effectors has been well documented in other fungal pathogens, most recently in another wheat pathogen *Zymospetoria tritici*, which is consistent with our findings [43]. Intrachromosomal translocation of *ToxA* was also described in *B. sorokinaina* [14] and highlights the mobility of *ToxA* within Ptr and *B. sorokinaina* via transposon activity and/or genomic recombination. Multiple HGT events were previously suggested for ToxA and its 14-kb surrounding region, the ToxhAT transposon [14]. In this study, we showed the nesting of ToxhAT in a larger mobile element Horizon which was not present in other ToxA containing isolates like BFP and M4. These findings support that ToxhAT may have been transferred to Ptr multiple times, with one HGT event nesting ToxhAT within Horizon.

### The ToxB gene is embedded on a large putative transposon in Ptr

For the first time, we showed the possible movement of *ToxB* as a multi-copy gene on a large 294-kb putative ‘Starship’ transposon dubbed ‘Icarus’. While ‘Icarus’ appears to lack the tyrosine recombinase ‘captain’, genes with DDE endonucleases are present where the captain is normally positioned. DDE endonucleases are associated DNA transposons transposases and are known to bind to TIRs which were identifiable at the edges of ‘Icarus’ (Fig. 5B) [33, 44]. This could indicate an alternative transposase associated with the mobility of ‘Icarus’. Alternatively, there appears to be a number of introgressed class I RNA transposons suggesting this putative Starship is rather old, having had the time to accumulate other transposons and expanded in size. The DDE endonucleases may also indicate the introgression of class II DNA transposons, and not be associated with Starship mobility. Combined with the fact that different Starship cargo genes are present within I-73–1 compared to D308 (NLR – HET, and DUF3723/phospholipase respectively) suggests that ‘Icarus’ has been disabled. No clear evidence of *ToxB* horizontal transfer has been found to date, but homologs of *ToxB* are present in closely and distantly related species (e.g. *P. bromi*, *Cochilobolus sativus*, *Alternaria alternata*, *Magnaporthe grisea*) [8]. It has been suggested that *ToxB* was acquired vertically from a common ascomycete ancestor [8, 15]. However, our discovery of *ToxB/toxb* on a large, potentially ancient, putative transposon inserted into an essential chromosome provides the new possibility of an alternative mechanism of acquisition via a horizontal transfer event older than that of *ToxA* (given the higher variability in *ToxB*/*toxb* reported sequences). Moreover, our limited analysis of DW-5 suggests a possible transposon associated with *ToxB* in that isolate. We found no co-linearity between the region containing *ToxB* in DW-5 and the reference isolates BFP or M4 and found no co-linearity with the putative transposon ‘Icarus’ from I-73–1 provides additional support of potential mobility of *ToxB* within Ptr or between species.

### CAZymes are an essential part of the Ptr genome

Ptr is a necrotrophic pathogen that possesses the ability to directly penetrate the host epidermal cells soon after spore germination [45], and this penetration is likely facilitated by the fungus ability to secrete cell wall degrading enzymes. The CAZymes and CAZy families identified in the Ptr pangenome support that Ptr is adapted for plant cell wall degradation, as > 30% of the total CAZome belongs to families involved in dismantling structural polysaccharides, such as cellulose, hemicelluloses, and the plant cuticle. Phytopathogenic fungi are known to deploy a number of CAZymes for invasion and infection [29, 31, 32]. As an example, *Fusarium culmorum* has been shown to degrade cellulose, xylans, and pectins during invasion of wheat spike tissues [46]. While previous work has linked the relatedness of CAZymes to fungal taxonomy [47], it is not known if there is functional specialization in different Ptr isolates. CAZomes may be tailored to accommodate unique structures in cell wall polysaccharides of different wheat varieties or plants growing in different geographic regions. Indeed, the diversification of AA9 structure, sequence, and expression levels in isolates of the phytopathogen *Rhizoctonia solani* has been proposed to be essential for the differential pathogenicity of these strains in rice and soybean [48], and the same may be true for Ptr and its hosts.

### Ptr has an open pangenome with a high accessory gene content

In an open pangenome, each added genome increases the number of accessory genes, while the number of core genes decreases [49]. In this work, we have shown that Ptr has an open pangenome. We also showed that Ptr, unlike other ascomycetes, contains a large proportion of accessory genes (57%) with a relatively small core gene set (43%). This is a significantly smaller core than the estimated 69% core previously reported in the pangenome of 11 Ptr isolates [17], and the 60% core estimated in the pangenome of the wheat pathogen *Z. tritici*, which was based on 19 isolates [50]. Our method of analysis assigns at most a single gene per isolate into a gene cluster [23, 51]; additionally, although the pipeline attempts to account for gene truncation, it is not always successful. For these two reasons, the number of accessory genes may be over-estimated relative to these other studies which used different grouping algorithms.

It has been suggested that plant pathogens possess accessory genomic elements related to pathogenicity [52]. However, we showed that the Ptr pangenome possesses a higher number of accessory genes in the non-pathogenic and weakly virulent isolates; these isolates also exhibited a larger proportion of orphan (singleton) genes. Approximately 70% of singleton genes had unknown functions, but perhaps these genes are involved in adaptation for a lifestyle divergent to wheat pathogenesis [25]. The open pangenome of Ptr may explain its ability to adapt to a wide variety of hosts, geographical regions, and environmental conditions. Despite its homothallic mating style, this pathogen evidently acquired virulence by the horizontal transfer of a large segment of DNA from other fungal species [7, 14]. The core genome is usually protected from high recombination and mutation rates in order to preserve its essential biological function, and the accessory genome is assumed to be under rapid evolution [52]. It is still unclear how the accessory genome in Ptr evolves and why the non-pathogenic isolates exhibited higher contents of accessory genes and TEs.

The observed separation of isolates based on ToxA production in the core protein phylogeny and the accessory protein hierarchical sets may be due to the host specialization of Ptr between bread and durum wheat. A similar observation has been made before, using simple sequence repeats on a similar collection of isolates [13]. The non-pathogenic isolates (except T-126–1 from Tunisia) were also distantly related to the rest of the isolates, and we observed a divergence in TE and effector content in these isolates, which is also an indicator of host specialization.

We also found variation in the genome size, particularly between pathogenic and non-pathogenic isolates. The genome size in the Dothideomycetes, based on the analysis of 101 species, varies tenfold: from less than 17 Mb to more than 177 Mb [53]. Our Ptr genome size based on the more accurate long-read assemblies averaged at 39.8 Mb, which was very consistent with previous genome size estimates for long-read Ptr in previous studies [17, 19, 35]. However, we showed that the non-pathogenic race 4 and weakly virulent race 5 isolates, G9-4 and 92-171R5 genome size were ~ 2 Mb larger than the average pathogenic isolates, and both isolates have an average of 3.1% more TE content than the other isolates. This clearly indicates an expansion of TEs in the genomes of these isolates. This larger genome size in non-pathogenic isolates was not expected, as previous reports of Ptr genome sizes indicated that pathogenic isolates have larger genomes, which was not consistent with our observations [17]. The larger genomes reported in pathogenic races/species relative to their non-pathogenic counterparts have been attributed to the presence of more repetitive elements, and it was posited that the need of pathogenic species to evolve in an arm’s race with its host could explain such genome expansion [54]. Indeed, in some plant pathogens, the acquisition of an entire supernumerary chromosome, often rich with repetitive sequences, by horizontal transfer is evident [55]. However, not all pathogenic species follow this trend of a larger genome [38]. A reduction in pathogen genome size may be an evolutionary mechanism to aid adaptation to specific environments or other niches and could explain the recent specialization of pathogenic species from related generalist non-pathogens [56–59]. Many pathogens adapt to exploit a limited number of species efficiently, and in some species, even a limited number of genotypes within a species [45] with rapid gene gains and losses previously linked to virulence adaptation in other studies [60, 61].

Nonetheless, while a larger genome size was found in the non-pathogenic race 4 isolate G9-4 and the weakly virulent race 5 isolate 92-171R5, this was not the case for the other race 4 isolates, 90–2 and T126-1, which had genome sizes similar to the pathogenic strains. As such, it is not easy to make a clear conclusion regarding genome size and pathogenicity in Ptr, and there is a need to explore additional non-pathogenic genomes. In previous studies, comparisons with Ptr non-pathogenic genomes were based on a single race 4 isolate (SD20-NP) [16, 17]. Additionally, in many other ascomycetes, the number of non-pathogenic genomes studied is limited in comparison with pathogenic genomes [62]. It is worth noting that non-pathogenicity in Ptr often has been assumed based on an isolate’s inability to cause disease symptoms on a limited number of host genotypes. However, what were assumed to be non-pathogenic race 4 isolates were recently found to cause extensive necrosis on specific durum wheat genotypes [63].

## Conclusions

Collectively, this work highlights the high plasticity and potential adaptability of Ptr as a global wheat pathogen. The large-scale chromosomal rearrangements, open pangenome, and the extensive accessory gene and TEs content of its genome reflect its cosmopolitan nature. In addition, the nesting of the virulence genes *ToxA* and *ToxB* within multiple transposon types is a significant indication of the rapid evolutionary nature of this pathogen and the contribution of transposons to virulence evolution and disease emergence.

## Methods

### Isolates, DNA extraction, and sequencing

Isolate details are provided in Table 1. Virulence phenotypes were previously confirmed [4, 12, 13, 64]. Isolates collected before 2016 and received from other labs were subjected to single spore isolates and their phenotypes confirmed on a wheat differential set as described by Aboukhaddour et al. (2013). Isolates were grown in 100 mL 1/4 concentration PDB and cultures incubated in a shaker (100 RPM) at room temperature (~ 25 °C), without light, until the fungal mat covered the surface of the medium. Fungal mats were harvested and washed twice using autoclaved mili Q water. Mats were placed in whirl pack bags on dry ice until freeze-dried. Dried mats were stored at − 20 °C until DNA extraction (no longer than 14 days). Genomic DNA was extracted using a ‘Genomic-tip 20/G’ kit (Qiagen) and sequenced with 150-bp paired-end, 400-bp inserts at 100 × coverage with Illumina HiSeq X. DNA from I-73–1 and D308 was extracted using a ‘Genomic-tip 100/G’ kit (Qiagen) and long-reads sequenced with PacBio RS II at 100 × coverage. All sequencing was performed by the Centre d’expertise et de services Génome Québec (Montreal, Canada). The reference isolate BFP (accession GCA_000149985) [16] was included in the pangenome analysis and used for full genome alignments. Isolates M4 (GCA_003171515) [17] and DW5 (GCA_003231415) [35] were also included for *Tox* gene and transposon analysis.

### De novo assemblies

Read quality was assessed with FASTQC [65] and poor-quality reads filtered. Reads were filtered using the standard Kraken2 database [66] and any reads tagged as non-fungi were omitted. A subset of isolates (90–2, AB88-2, ASC1, AZ35-5, and I-72–1) were selected to test assembly program suitability using the Illumina reads. The assemblers were Shovill with SPAdes [67, 68], Shovill with MEGAHIT [69], SOAPDenovo2 [70], and CLC Genomics Workbench 12 (Qiagen) all program arguments available in the GitHub repository. QUAST [71] output and BUSCO scores were used to assess assembly quality. All remaining short reads were assembled with Shovill/SPAdes. Long reads were assembled with Flye [72] and polished with short-read data using Pilon [73]. Completeness of the long-read assemblies was also assessed by the alignment of raw reads (Additional file 9).

### Gene annotations

FunGAP (v1.0.1) is an annotation pipeline specifically for fungi [74]. FunGAP makes use of many programs: RepeatModeler [75], RepeatMasker [76], HISAT [77], Trinity [78], Augustus [79], MAKER [80], BRAKER [81], InterProScan [82], and BLAST + [83]. MAKER and BRAKER utilize RNA-seq reads. RNA-seq reads from vegetative Ptr mycelia were retrieved from BioPlatforms Data Portal (https://data.bioplatforms.com/; Sample ID: 102.100.100.14350) [17]. FunGAP provides a script to select a prediction model for Augustus, with *Botrytis cinerea* selected as the model in this case. Default settings provided by FunGAP were used to annotate all assemblies. After pangenome analysis (discussed below), representatives from the core and accessory gene clusters were used for functional annotation with Pfam v28.0 [84]. All predicted gene sets were assessed for completeness using the Ascomycota BUSCO gene set (odb9) [85, 86].

### Pangenome analysis

Pangloss is a pangenome pipeline designed for microbial eukaryotes like fungi [23]. Panoct [51] is the primary program for calculating a pangenome. A custom bash script was used to modify *.gff3 files from FunGAP into *.attributes format required by PanOCT. Output from all-vs-all BLASTp (E10^−4^) was used to determine if a gene belongs in the core or accessory genome. Pangloss does not distinguish singletons from the accessory genome, a custom bash script separated singletons. Singletons were run through Pangloss for a second iteration. Binary gene presence or absence tables were parsed to generate figures showing the percentage of the genome comprised by core, accessory or singleton genes, total genes in the pangenome as new genomes were added, and the number of genes in accessory clusters. Statistics including *t*-tests, ANOVA, and Tukey’s HSD were performed with R (v3.4.3) in RStudio.

### CAZymes and effectors

Phobius v1.01 (with –short) [87] was used to filter amino acid sequences for signal peptides and transmembrane domains. Sequences with signal peptides and without transmembrane domains were used as input for EffectorP-2.0 [88]. Genes identified as potential effectors (probability > 50%) were extracted from gene sets and used as input for Pangloss to assess the presence/absence between isolates. Predicted protein sequences produced by FunGap from all isolates were annotated by dbCAN2 [89] and manually curated for selected GH, CE, PL, and AA families relevant for plant cell wall degradation from the CAZy database [27].

### Phylogeny and accessory sets

Individual alignments for each of the 10,159 amino acid core orthologue clusters were performed using MUSCLE (v3.6) (this may include multi-copy genes which would have been treated individually) [90]. Bash and python scripts were used to concatenate and combine the aligned core amino acid gene set for each isolate creating a single alignment. The core alignment was input for RAxML [91] to generate a maximum likelihood (ML) phylogeny using the PROTGAMMA model, 1000 bootstrap replicates, and a starting seed of 10. Variant call files were generated via GATK HaplotypeCaller [92] using BFP as a reference. SNPs were converted to fasta format with vcf2phylip [93] and input into RAxML for a ML tree using the GRTCAT model. The ML trees were visualized with FigTree [94] and the Tox/location content added manually. Binary data was filtered to include only accessory genes and was input for the R package HierarchicalSets with default settings [95].

### Transposable element content

Transposable elements (TEs) were identified and categorized using EDTA with the higher sensitivity setting (–sensitive 1) [96], which utilizes several programs: LTRharvest [97], LTR_FINDER [98], LTR_FINDER_parrallel [99], LTR_retriever [100], TIR-Learner [101], Generic Repeat Finder [102], HelitronScanner [103], and TEsorter [104]. Output was aggregated and the total TE content per genome (stacked histogram) and TE content as a function of genome size (Mb) visualized.

### Genome organization

Pair-wise full genome alignments of long-read assemblies: I-73–1, D308, BFP, M4 (1.1), and DW-5 were performed with minimap2 [105] and visualized with dotPlotly [106]. For chromosomes with *ToxA* and *ToxB* putative transposons, syntenic blocks with a minimum length of 6500 bp were generated using Sibelia (v3.0.7) [107], then visualized using Circos (v0.69–8) [108]. Selected proteins within the putative transposons were modelled with Phyre2 [26]. Mauve [109] alignments were used to identify target sites and target site duplications for transposons associated with *ToxA* and *ToxB/toxb*. Gene annotations (gff3), functional annotations (pfam), and BLAST outputs were used to manually construct the transposon schematics. Intergenic distances were calculated from gff3 files using an adapted R-script [110] and plotted using a hexagonal heatmap of 2d bin counts geom_hex with 50 bins. Potential GDR and GSR genes were selected based on the 90th percentiles of ITL distances, with potential GDR genes having 5′ and 3′ ITLs < 2070 bp and GSR genes having 5′ and 3′ ITLs > 7394 bp. GDR and GSR genes were codon aligned to homologs of BFP using PRANK [111, 112] with some sequences being reverse complimented prior to alignment. Alignments were assessed for evolution rates via dN/dS ratios using codeml from the PAML package [113]. dN/dS ratios were subject to unpaired Welch two-sample *t*-test assuming unequal variance to test significant differences. Long-read genomes were uploaded to RIPper [114] to assess RIP and LRAR content. The core vs. accessory nature of contigs was assessed by alignment (i.e. presence in all isolates) and evaluation of the ratio of core to accessory genes, percentage of total genes, and genes per Mb on each contig.

## Supplementary Information

**Additional file 1. **Genes were clustered based on the number of isolates in which they were present (e.g. genes in cluster 2 are present in two isolates, genes in cluster 3 are present in three isolates, etc.). Genes in low clusters may represent recently gained genes, as only a few isolates contain them, while genes in high clusters may represent recently lost genes, as most isolates contain them. Clusters 1 and 41 were omitted as they represent singletons and the core gene set respectively.

**Additional file 2. **Maximum likelihood phylogenies created by RAxML based on SNP data. **a** ML tree with all isolates; **b** ML tree with the divergent outgroup omitted to aid reading of the other branches.

**Additional file 3. **Curated output from dbCAN for grouping and counting the number of genes in specific CAZyme families.

**Additional file 4. **Annotation of genes present within the 143 kb *Starship* transposon ‘Horizon’ present in the race 8 isolate I-73-1.

**Additional file 5. **Circular alignments of *ToxB* carrying contigs. **a** contig 5 from race 3 isolate D308, contig 12 from race 8 isolates I-73-1, and contig NQIK01000005 from race 1 isolates M4. A large 294 Kb region which contains three copies of the *ToxB* (black arrow) is visible which aligns with a section in D308 containing a single copy of the inactive *toxb *(grey arrow). **b** DW-5 contigs (CM025819.1 and CM025824.1) align to Pt-1C-BFP chromosomes (chr 5 and 11 respectively). Sections containing *ToxB* (black arrows) do not appear to be co-linear with each other or the reference chromosomes indicating possible transposon activity. These segments of DW-5 do not appear to align with ‘Icarus’ from I-73-1 or D308.

**Additional file 6. **Number of core, accessory, and singleton genes on individual contigs of the long-read assemblies of I-73-1 and D308.

**Additional file 7. **Density of ITL sizes for I-73-1, red bars indicate 90^th^ percentile cut-off values.

**Additional file 8. **Intergenic distances of all genes in* Pyrenophora tritici-repentis* isolate D308. The 3’ intergenic length (x-axis) is the distance (bp) from the 3’ end of current gene to the 5’ end of next, and the 5’ intergenic length (y-axis) is the distance from the 3’ end of the previous gene to the 5’ end of the current gene.

**Additional file 9. **Alignment of raw read data to long-read assemblies; **a** I-73-1; **b** D308.

**Additional file 10. **List of GenBank accession numbers associated with the genomes generated by this study and of the genomes downloaded and used for comparison.

## Data Availability

Isolate accession numbers are available in Additional file 10. Raw data is available under NCBI BioProject PRJNA803191 [115]. Scripts, program settings, and additional files from this work are available at https://github.com/fungal-spore/Ptr-pangenome-paper [116].
