# Supplementary figures and images for: The pangenome of the wheat pathogen Pyrenophora tritici-repentis reveals novel transposons associated with necrotrophic effectors ToxA and ToxB
Source: BMC Biol. 2022 Oct 24;20:239. doi: 10.1186/s12915-022-01433-w (PMC9594970; doi:10.1186/s12915-022-01433-w)

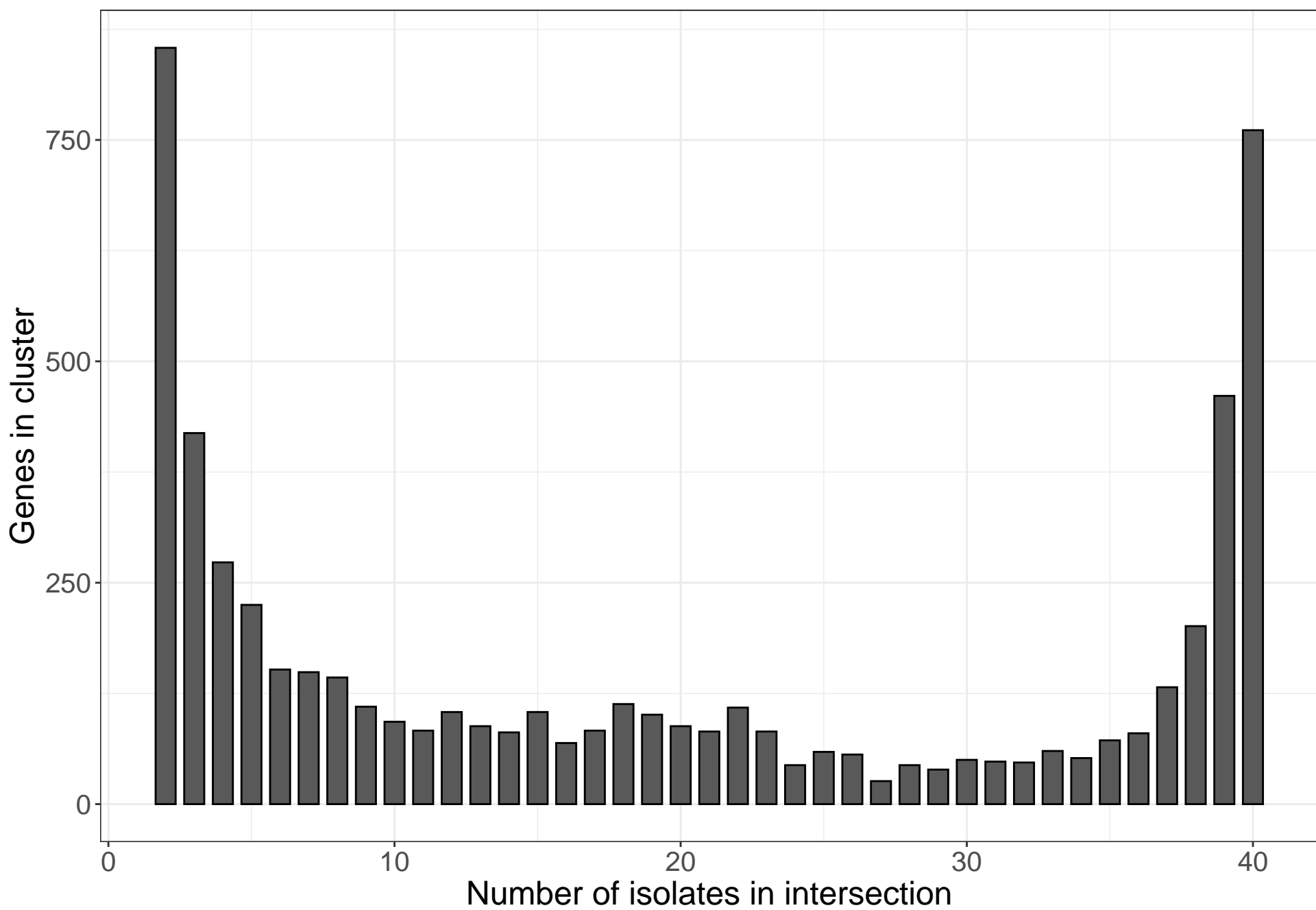

Supplement: Supplementary file 1 — Additional file 1. Genes were clustered based on the number of isolates in which they were present (e.g. genes in cluster 2 are present in two isolates, genes in cluster 3 are present in three isolates, etc.). Genes in low clusters may represent recently gained genes, as only a few isolates contain them, while genes in high clusters may represent recently lost genes, as most isolates contain them. Clusters 1 and 41 were omitted as they represent singletons and the core gene set respectively. [file 12915_2022_1433_MOESM1_ESM.pdf]

a

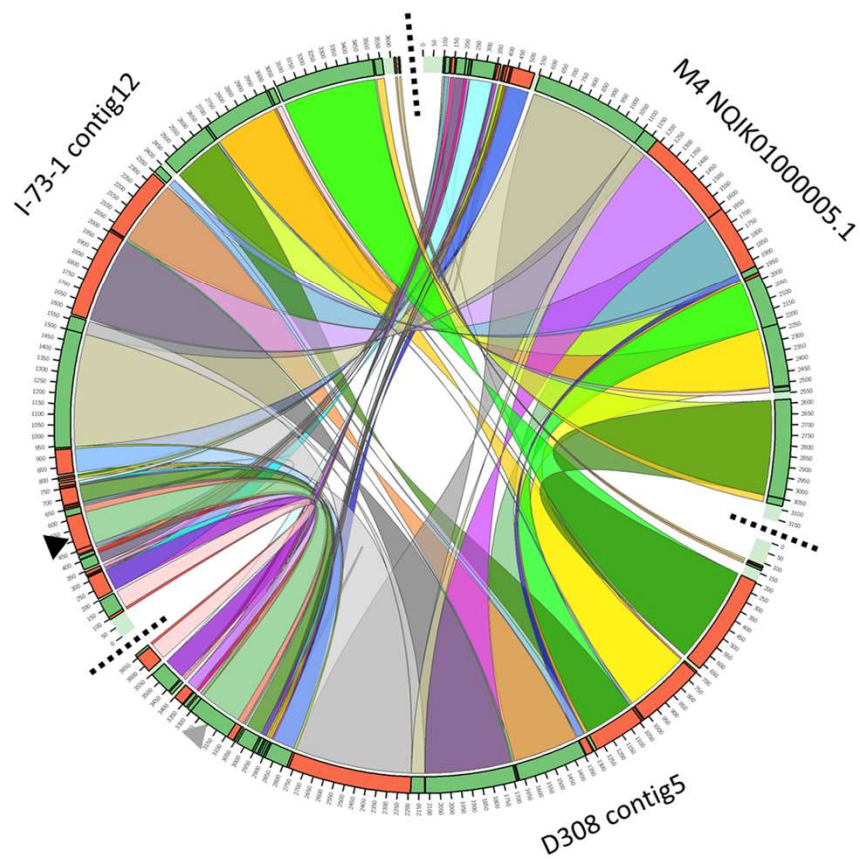

b

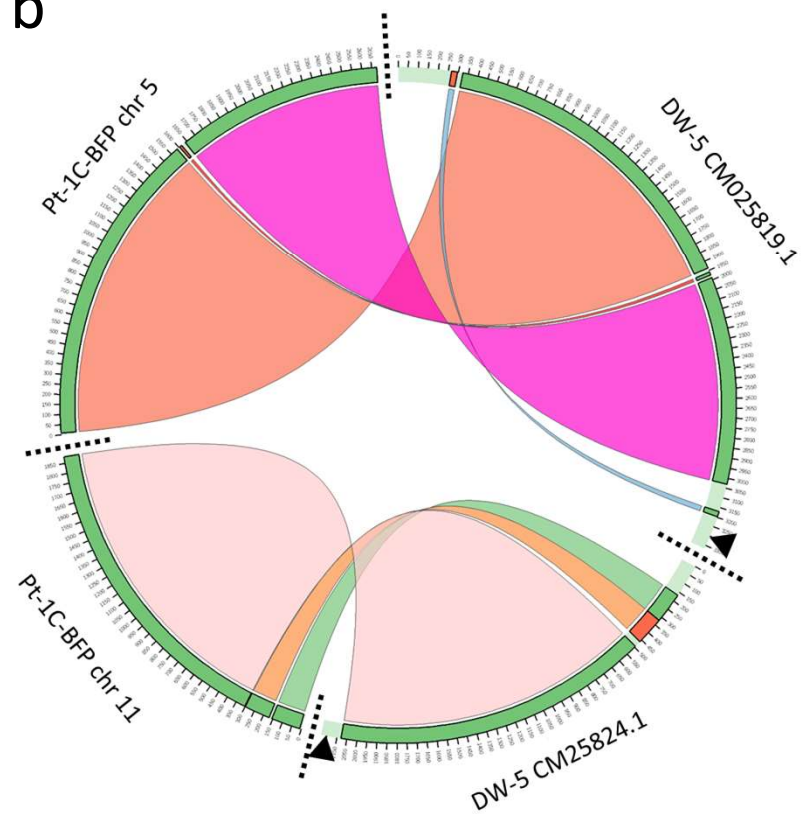

Supplement: Supplementary file 5 — Additional file 5. Circular alignments of ToxB carrying contigs. a contig 5 from race 3 isolate D308, contig 12 from race 8 isolates I-73-1, and contig NQIK01000005 from race 1 isolates M4. A large 294 Kb region which contains three copies of the ToxB (black arrow) is visible which aligns with a section in D308 containing a single copy of the inactive toxb (grey arrow). b DW-5 contigs (CM025819.1 and CM025824.1) align to Pt-1C-BFP chromosomes (chr 5 and 11 respectively). Sections containing ToxB (black arrows) do not appear to be co-linear with each other or the reference chromosomes indicating possible transposon activity. These segments of DW-5 do not appear to align with ‘Icarus’ from I-73-1 or D308. [file 12915_2022_1433_MOESM5_ESM.pdf]

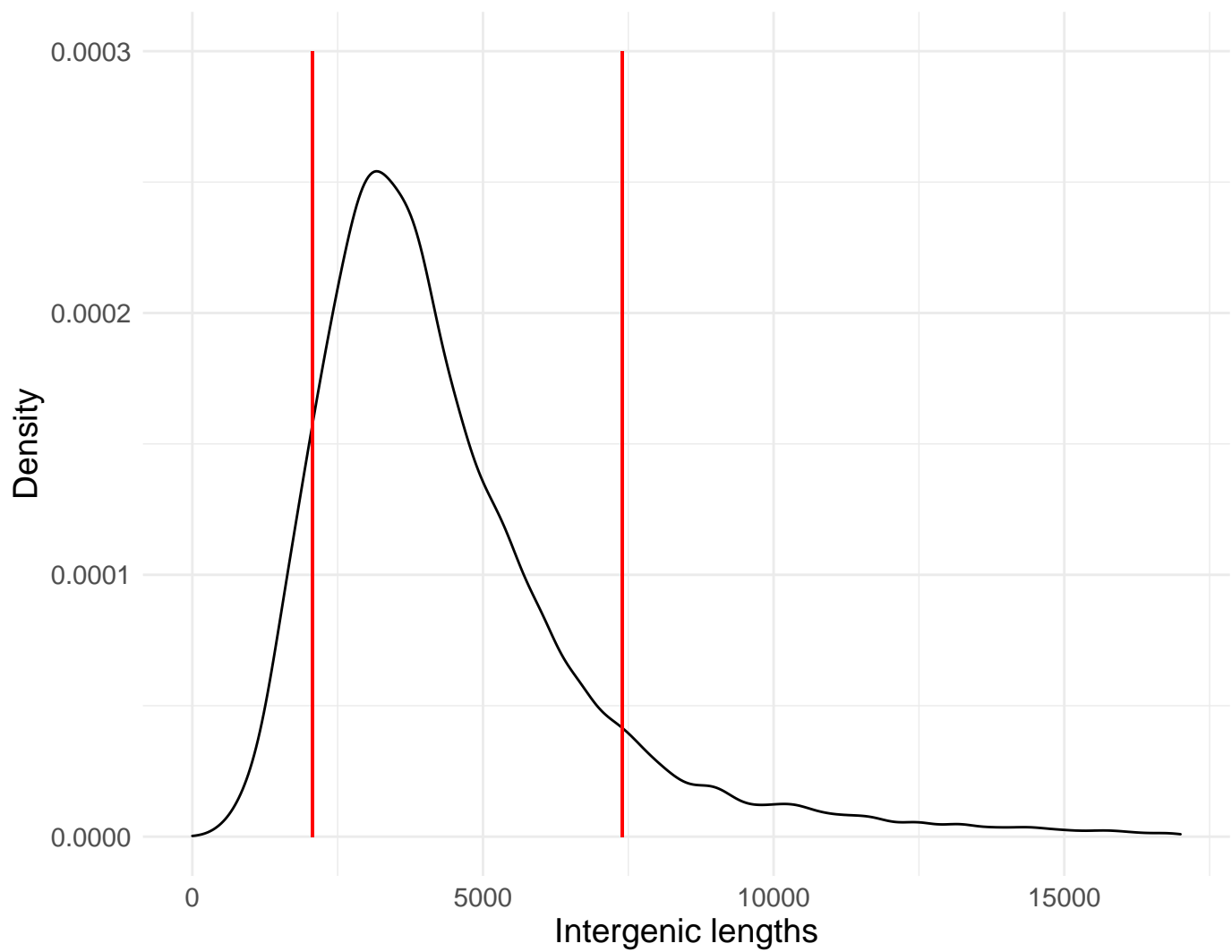

Supplement: Supplementary file 7 — Additional file 7. Density of ITL sizes for I-73-1, red bars indicate 90th percentile cut-off values. [file 12915_2022_1433_MOESM7_ESM.pdf]

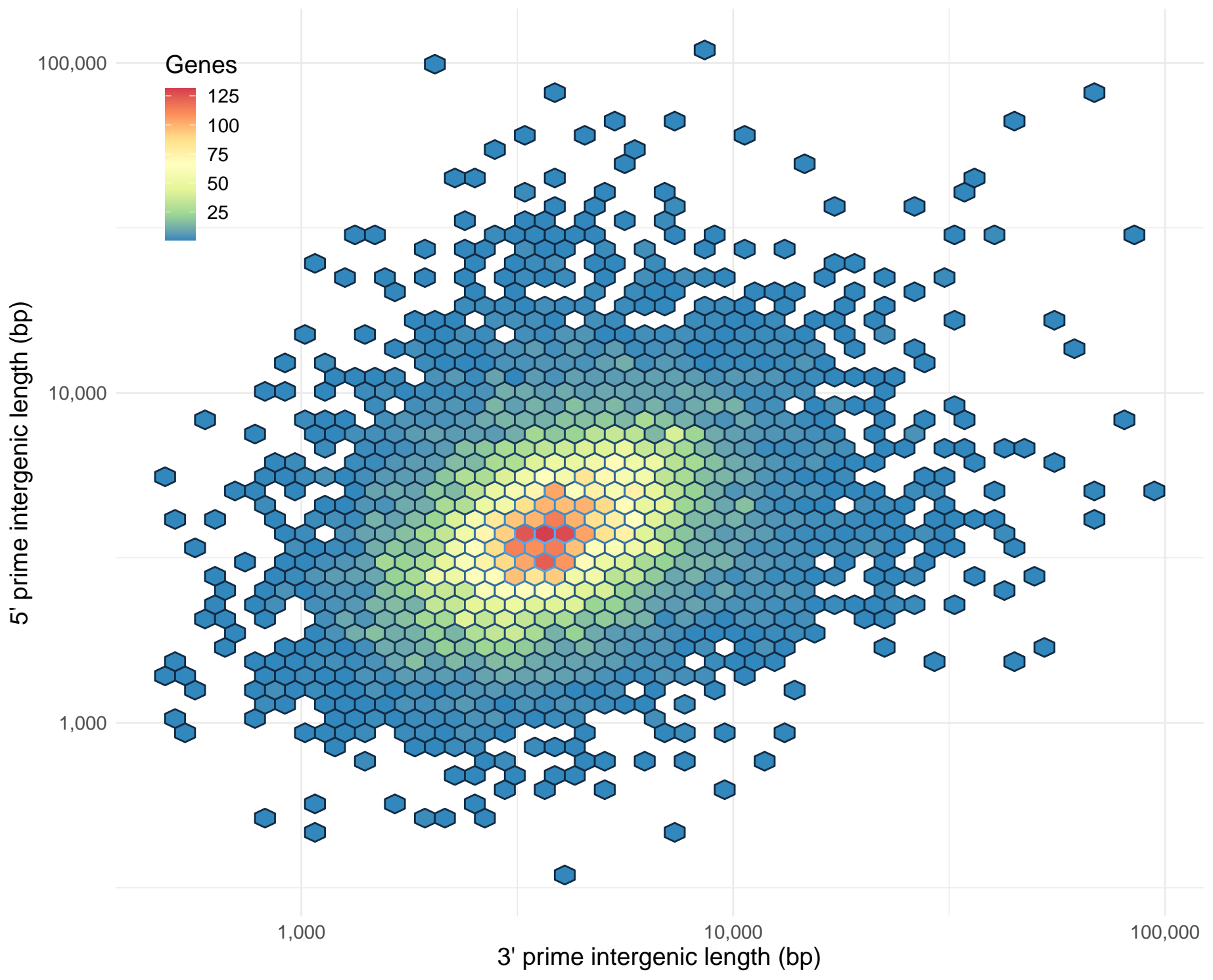

Supplement: Supplementary file 8 — Additional file 8. Intergenic distances of all genes in Pyrenophora tritici-repentis isolate D308. The 3’ intergenic length (x-axis) is the distance (bp) from the 3’ end of current gene to the 5’ end of next, and the 5’ intergenic length (y-axis) is the distance from the 3’ end of the previous gene to the 5’ end of the current gene. [file 12915_2022_1433_MOESM8_ESM.pdf]

a

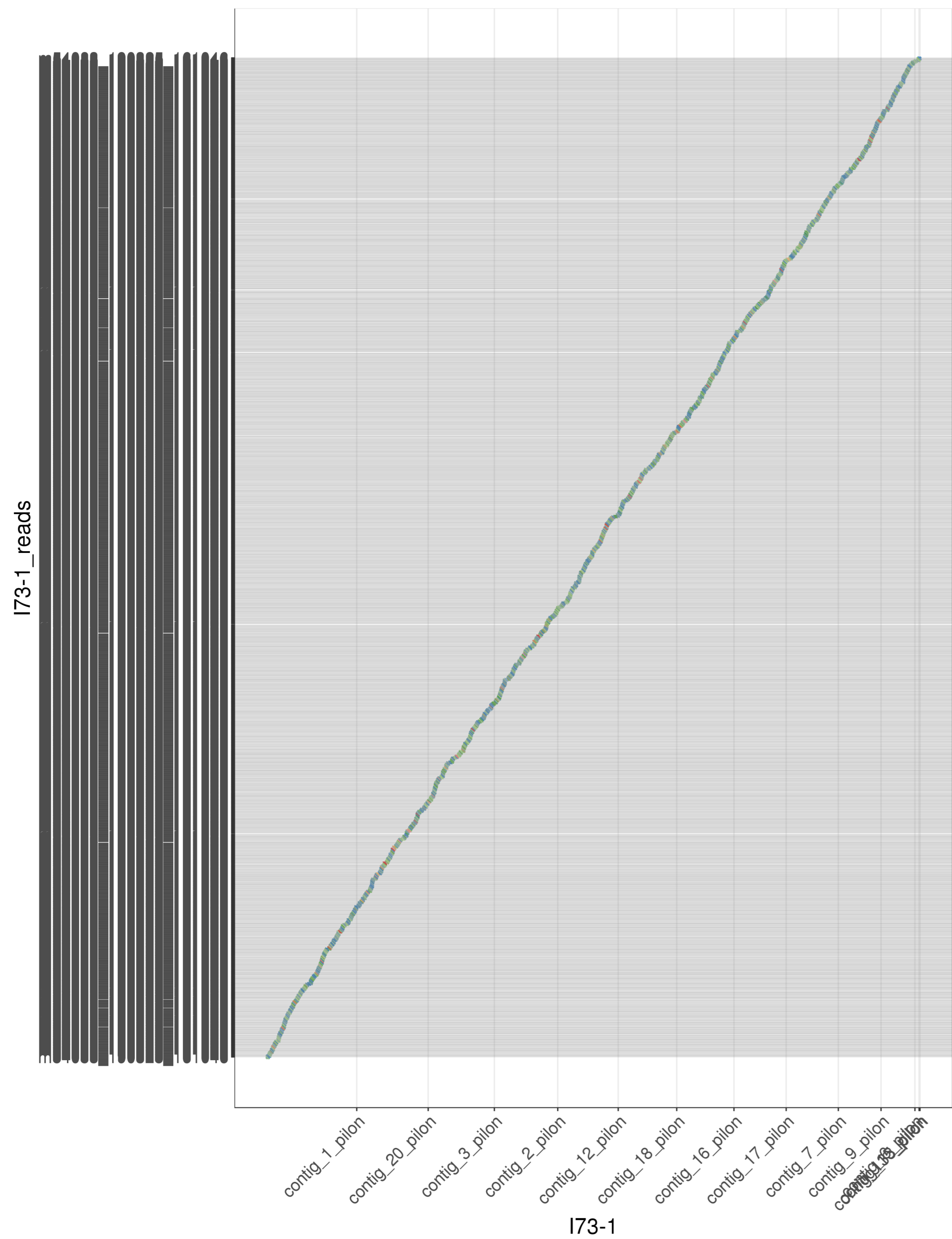

b

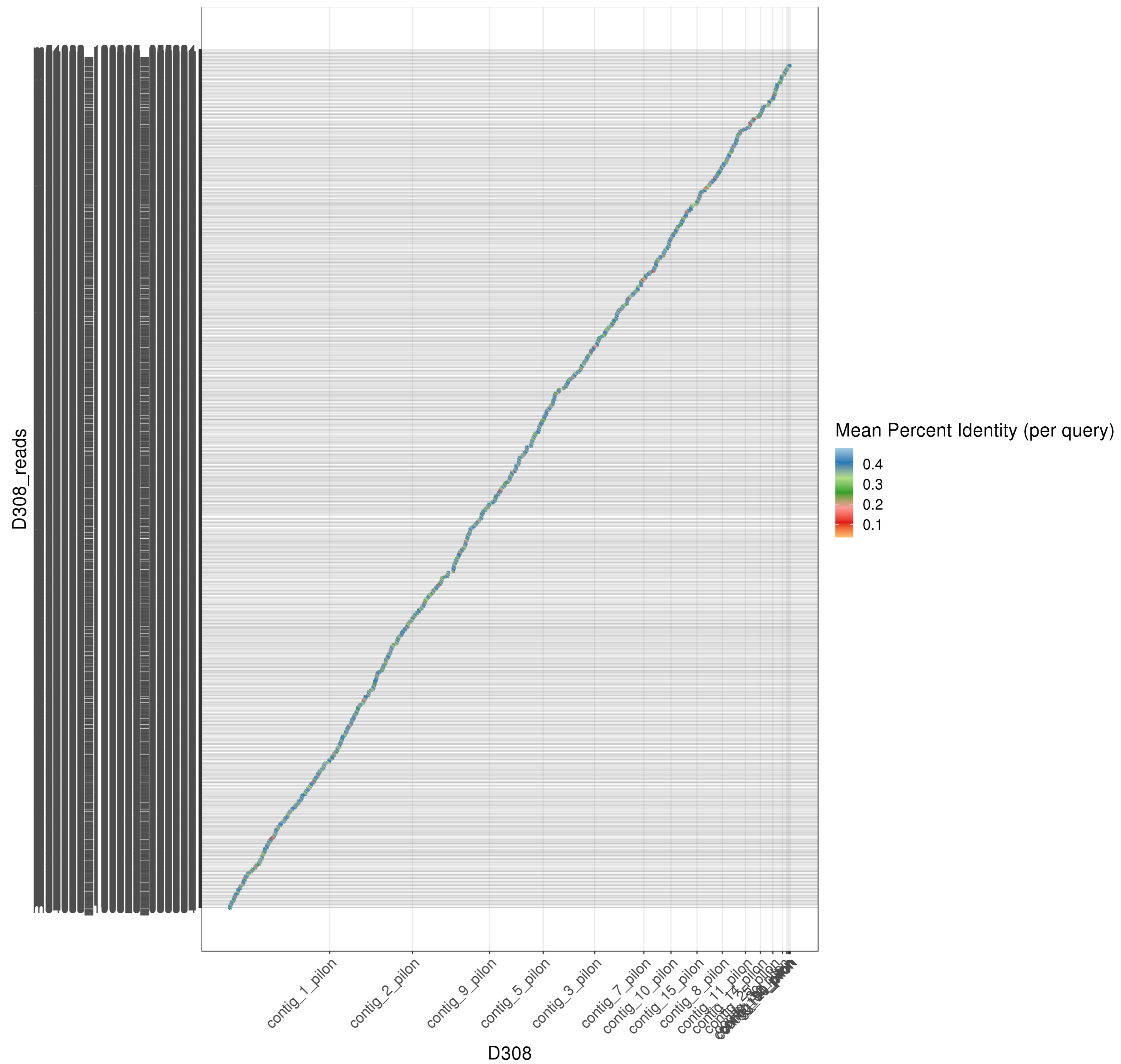

Supplement: Supplementary file 9 — Additional file 9. Alignment of raw read data to long-read assemblies; a I-73-1; b D308. [file 12915_2022_1433_MOESM9_ESM.pdf]
