# Supplementary material for: The pangenome of the wheat pathogen Pyrenophora tritici-repentis reveals novel transposons associated with necrotrophic effectors ToxA and ToxB
Source: BMC Biol. 2022 Oct 24;20:239. doi: 10.1186/s12915-022-01433-w (PMC9594970; doi:10.1186/s12915-022-01433-w)
Supplement: Supplementary file 2 — Additional file 2. Maximum likelihood phylogenies created by RAxML based on SNP data. a ML tree with all isolates; b ML tree with the divergent outgroup omitted to aid reading of the other branches. [file 12915_2022_1433_MOESM2_ESM.pdf]

a

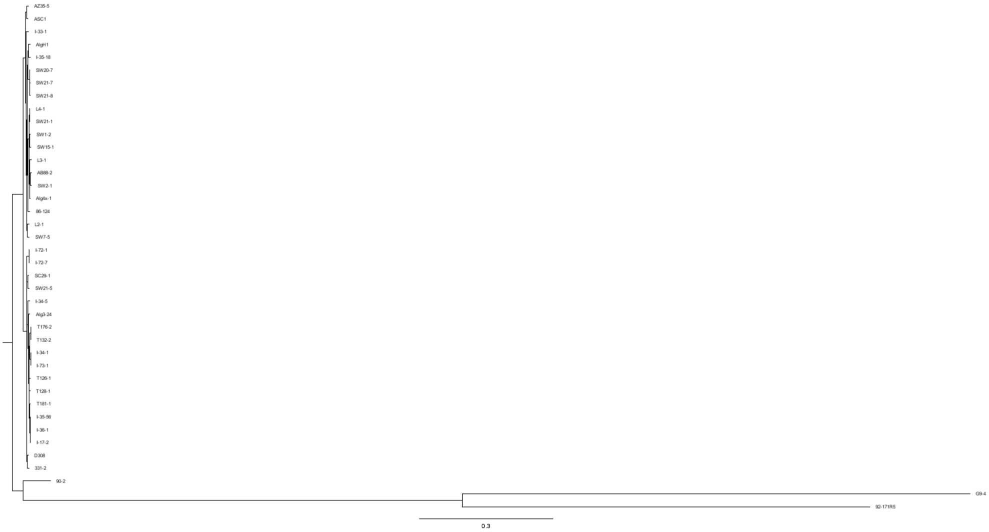
AZ35-5
ASC1
I-33-1
AlgH1
I-35-18
SW20-7
SW21-7
SW21-8
L4-1
SW21-1
SW1-2
SW15-1
L3-1
AB88-2
SW2-1
Alg4x-1
86-124
L2-1
SW7-5
I-72-1
I-72-7
SC29-1
SW21-5
I-34-5
Alg3-24
T176-2
T132-2
I-34-1
I-73-1
T126-1
T128-1
T181-1
I-35-56
I-36-1
I-17-2
D308
331-2
90-2
G9-4
92-171R5
0.3

b

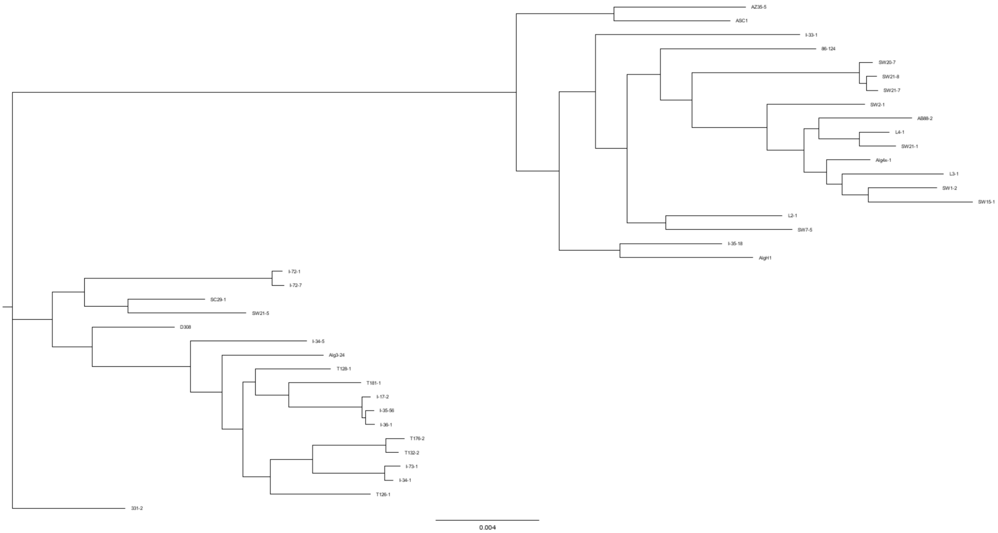
AZ35-5
ASC1
I-33-1
86-124
SW20-7
SW21-8
SW21-7
SW2-1
AB88-2
L4-1
SW21-1
Alg4x-1
L3-1
SW1-2
SW15-1
L2-1
SW7-5
I-35-18
AlgH1
I-72-1
I-72-7
SC29-1
SW21-5
D308
I-34-5
Alg3-24
T128-1
T181-1
I-17-2
I-35-56
I-36-1
T176-2
T132-2
I-73-1
I-34-1
T126-1
331-2
0.004
